# Loss of starch synthase IIIa changes starch molecular structure and granule morphology in grains of hexaploid bread wheat

**DOI:** 10.1038/s41598-022-14995-0

**Published:** 2022-06-25

**Authors:** Brendan Fahy, Oscar Gonzalez, George M. Savva, Jennifer H. Ahn-Jarvis, Frederick J. Warren, Jack Dunn, Alison Lovegrove, Brittany A. Hazard

**Affiliations:** 1 John Innes Centre, Norwich, UK; 2 Quadram Institute Bioscience, Norwich Research Park, Norwich, NR4 7UQ UK; 3 Rothamsted Research, Harpenden, UK

**Keywords:** Agricultural genetics, Plant biotechnology, Molecular engineering in plants, Plant breeding, Plant genetics, Plant molecular biology, Plant sciences

## Abstract

Starch synthase III plays a key role in starch biosynthesis and is highly expressed in developing wheat grains. To understand the contribution of SSIII to starch and grain properties, we developed wheat *ssIIIa* mutants in the elite cultivar Cadenza using in silico TILLING in a mutagenized population. SSIIIa protein was undetectable by immunoblot analysis in triple *ssIIIa* mutants carrying mutations in each homoeologous copy of *ssIIIa* (A, B and D). Loss of SSIIIa in triple mutants led to significant changes in starch phenotype including smaller A-type granules and altered granule morphology. Starch chain-length distributions of double and triple mutants indicated greater levels of amylose than sibling controls (33.8% of starch in triple mutants, and 29.3% in double mutants vs. 25.5% in sibling controls) and fewer long amylopectin chains. Wholemeal flour of triple mutants had more resistant starch (6.0% vs. 2.9% in sibling controls) and greater levels of non-starch polysaccharides; the grains appeared shrunken and weighed ~ 11% less than the sibling control which was partially explained by loss in starch content. Interestingly, our study revealed gene dosage effects which could be useful for fine-tuning starch properties in wheat breeding applications while minimizing impact on grain weight and quality.

## Introduction

Wheat (*Triticum aestivum*) is a staple cereal crop that provides nearly 20% of the calories consumed worldwide^1^. Although wheat is a valuable source of nutrition, there is growing concern about the effects of wheat-based foods on health including the rising incidence of obesity and type 2 diabetes^2,3^. Most wheat food products are made from refined white flour which is mainly comprised of starch (~ 85% dry weight); they tend to be low in fibre and are rapidly digested in the upper gastrointestinal tract^3,4^. Thus, elevating the levels of resistant starch, an important component of dietary fibre, is a focus of many wheat improvement programs.

The major component of the wheat grain is starch, a mixture of two glucose polymers, amylose (~ 20 to 30%) and amylopectin (~ 70 to 80%), which differ in size, glucose chain-length, and degree of branching^5^. Amylose polymers are mostly linear, consisting of α-1,4 linked glucose chains with occasional α-1,6 bonds forming branch points, while amylopectin polymers are large and highly branched (with 4–5% of glucosyl residues carrying branch points). Starch polymers form granules in the amyloplasts of endosperm cells in developing wheat grains. The granules have a highly organized structure with amorphous lamellae that contain amylose polymers and branch points of amylopectin and crystalline lamellae that form from the ordered packing of short amylopectin chains^6^.

Three types of enzymes are required for amylose and amylopectin synthesis from adenosine diphosphate glucose (ADP-glucose) which is made in the endosperm from sucrose produced during photosynthesis and transported to the developing grain: soluble starch synthases and granule-bound starch synthases (SSs and GBSSs) which elongate glucose chains, starch branching enzymes (SBEs) which introduce branch points, and starch debranching enzymes (DBEs) which trim branched chains to create a structure that can crystallize to form the granule matrix^7^. *Starch Synthase IIIa* (*SSIIIa*) is strongly expressed in developing wheat endosperm cells and thus likely to make a major contribution to starch synthesis^8^.

Resistant starch is a type of starch that resists digestion in the small intestine and reaches the colon where it is further metabolized by gut microbes^9^. Growing evidence suggests resistant starch is associated with health benefits such as reduced glycaemic response, thus elevating the resistant starch content of wheat grains has potential for improving their nutritional value^3,9^. For example, recent studies of wheat bread and pasta with resistant starch levels ranging from ~ 7 to 9% led to reductions in postprandial glycaemic response in vivo^10,11^. Starches with high levels of amylose tend to have a greater content of resistant starch than those with low amylose content, so a key strategy to increase resistant starch in wheat is to develop varieties that produce high-amylose starch^12^. Amylose and resistant starch levels can be elevated in wheat by combining induced mutations in *SSIIa* or *SBEII* genes^13–17^. However, initial analyses of some mutants have revealed adverse effects on yield components including grain weight and on grain quality characteristics, so it is not yet clear if these mutants can be used to develop commercially viable wheat lines^18,19^. Therefore, targeting other key starch biosynthesis genes might provide a better route to resistant starch.

A possible candidate gene is *SSIII.* Studies of *ssIIIa* mutants in potato, rice and maize and of SSIII in vitro have suggested it has a key role in synthesis of long amylopectin chains (reviewed in Nakamura^7^). Manipulation of *SSIII* genes affects amylose and resistant starch content in other cereal species. For example, *dull1* maize mutants deficient in starch synthase III have higher amylose contents than wild-type controls (25.4–30.2% vs. 21.5% in wildtype)^20^. Similarly, the barley *amo1* mutant has higher levels of amylose relative to a wild-type control (15.0 mg per grain vs. 10.7 mg per grain in the wild-type control, a relative increase of 40%)^21^. Furthermore, a mutation in a *SSIIIa* gene confers high levels of resistant starch in cooked rice (~ 6.0% vs. ~ 1.5% in wild-type controls)^22^. There is also evidence that manipulation of *SSIIIa* in rice can influence starch structure indirectly, through pleiotropic effects on activities of other starch-synthesizing enzymes including the amylose-synthesizing enzyme GBSS^23^. Likewise, the lack of SSIII in maize leads to a decrease in starch branching enzyme IIa activity and increased starch synthase I activity^24,25^. The reported pleiotropic effects of loss of SSIII are unsurprising given the evidence that starch biosynthetic enzymes work together in complexes and that their activities are likely coordinated rather than independent^26^. For wheat, there is no peer-reviewed literature on the impact of loss of *ssIIIa* on starch accumulation and properties. Information in a patent application indicates that combined mutations in wheat *ssIIIa* homoeologues can lead to elevated levels of amylose and components of dietary fibre^27^.

Elucidating the roles of starch-related genes in wheat presents challenges in identifying loss-of-function mutations, which are likely to be masked by functional gene copies found in the other homoeologous genomes^28^. However, the recent advances in wheat genomics resources, specifically the exome capture and re-sequencing of wheat TILLING (Targeting Induced Local Lesions IN Genomes) mutants, allow induced mutations in different homoeologues and genes to be combined by crossing wheat mutants^29^. The functions and interactions of individual genes and homoeologues can then be investigated.

To discover whether manipulation of SSIIIa expression can alter starch properties and elevate levels of resistant starch in wheat we have generated a novel set of mutants in the elite bread wheat cultivar Cadenza carrying combinations of null mutations in *SSIIIa* homoeologues. Here we describe the development and characterization of the starch and grain properties of the *ssIIIa* TILLING mutants.

## Results

### In silico expression analysis of *SSIIIa* and *SSIIIb* genes

Genomic DNA sequences for *SSIIIa* and *SSIIIb* homoeologues in the elite bread wheat cultivar Cadenza were identified using the Grassroot Genomics BLAST Search (https://grassroots.tools/service/blast-blastn) to find homologous sequence to the *SSIII* genomic DNA sequence previously isolated by Li et al.^8^ (GenBank accession no. AF258609). Using the Wheat Expression Browser (http://www.wheat-expression.com/) in silico expression profiling of *SSIIIa* and *SSIIIb* paralogs and homoeologues was performed to investigate expression patterns for distinct plant tissues and for different stages during grain development. A heat-map comparing expression of *SSIIIa* and *SSIIIb* indicated that expression of *SSIIIa* is mainly limited to the grain, whereas *SSIIIb* is expressed in the roots, leaves/shoots, and spike as well as the grain (Fig. S1). *SSIIIa* transcript is highest around 10 and 15 days post anthesis, which is consistent with the mid-development peak of *SSIII* transcript observed in RNA isolated from developing wheat endosperms by Li et al.^8^. Comparisons of *SSIIIa* and *SSIIIb* transcript profiles suggest this peak was almost entirely due to *SSIIIa*; *SSIIIb* has high expression very early in development (~ 2 days post anthesis) but lower expression throughout the rest of development. Given these results we supposed that *SSIIIa* is the predominant form expressed in the grain and corresponds to the cDNA and genomic DNA sequences previously isolated by Li et al.^8^. In light of these data we focused on developing mutants for *SSIIIa* homoeologues which we presumed would have the greatest impact on starch properties in the grain.

### TILLING for *ssIIIa* genes

Mutations causing premature stop codons in the A, B and D genome copies of *SSIIIa* (*ssIIIa*-A, *ssIIIa*-B, *ssIIIa*-D) (Table 1 and Fig. S2) were identified using the in-silico Wheat TILLING resource for the cultivar Cadenza (http://www.wheat-tilling.com/). To predict the effect of the mutations on protein function we used the reference SSIII amino acid sequence (1628 residues) from a hexaploid wheat *SSIII* cDNA clone (GenBank accession AF258608), and previously defined protein domain regions described by Li et al.^8^. Each of our selected mutations was predicted to result in a protein truncated before the catalytic domain or within it, rendering the mutant protein inactive.

**Table 1:** Selected mutations for SSIIIa homoeologues.

Gene	Line	Codon	Protein coordinate and predicted effect	Chrom	Exon	Protein domain
*SSIIIa-A*	C2074	Cag → Tag	Q 531 (stop codon)	1A	3	Variable repeat region
*SSIIIa-B*	C0905	Caa → Taa	Q 1306 (stop codon)	1B	10	Catalytic domain
*SSIIIa-D*	C0291	Cga → Tga	R 435 (stop codon)	1D	3	Variable repeat region

Line is the mutant number in the TILLING population (e.g., C2074 = Cadenza 2074). Codon is the sequence of the control (left) and mutant (right) codons; the uppercase letter indicates which base was mutated. Protein coordinate includes the affected amino acid reside and the position based on deduced amino acid sequences from SSIIIa genomic DNA in the Cadenza cultivar. Chrom is the homoeologous chromosome. Protein domain is based on the defined proteins domains described in Li et al.^8^

### Generation of *ssIIIa* mutants

The TILLING mutants for each homoeologue of *ssIIIa* (*ssIIIa*-A, *ssIIIa*-B, *ssIIIa*-D) were crossed to create a line lacking active SSIIIa protein. Plants heterozygous for mutations in all three *ssIIIa* homoeologues were self-fertilized to produce segregating F_2_ populations. Using KASP markers (Table S1), 724 segregating F_2_ plants were screened for homozygous single, double and triple mutants (single: *ssIIIa*-A, *ssIIIa*-B, *ssIIIa*-D; double: *ssIIIa*-AB, *ssIIIa*-AD, *ssIIIa*-BD; triple: *ssIIIa*-ABD). The number of plants selected for each genotype class is included in Table S2. At least eight plants were selected for each genotype class, except for the double mutant *ssIIIa*-AD for which only five plants were identified. Despite fewer *ssIIIa*-AD plants, a Chi-square test showed no evidence of segregation distortion (*P* = 0.8034). In addition to mutant genotypes, siblings without mutations in *SSIIIa* were selected to provide an ideal control; these lines are expected to have the same level of segregating background mutations left over from the TILLING process as the *ssIIIa* mutants. Genome-specific sequencing primers were designed to validate the selected mutations (Table S3) and all triple mutant and sibling control plants were sequenced to confirm presence or absence of each mutant allele (*ssIIIa*-A, *ssIIIa*-B, and *ssIIIa*-D).

### Immunoblot analysis of SSIIIa

**Figure 1:**
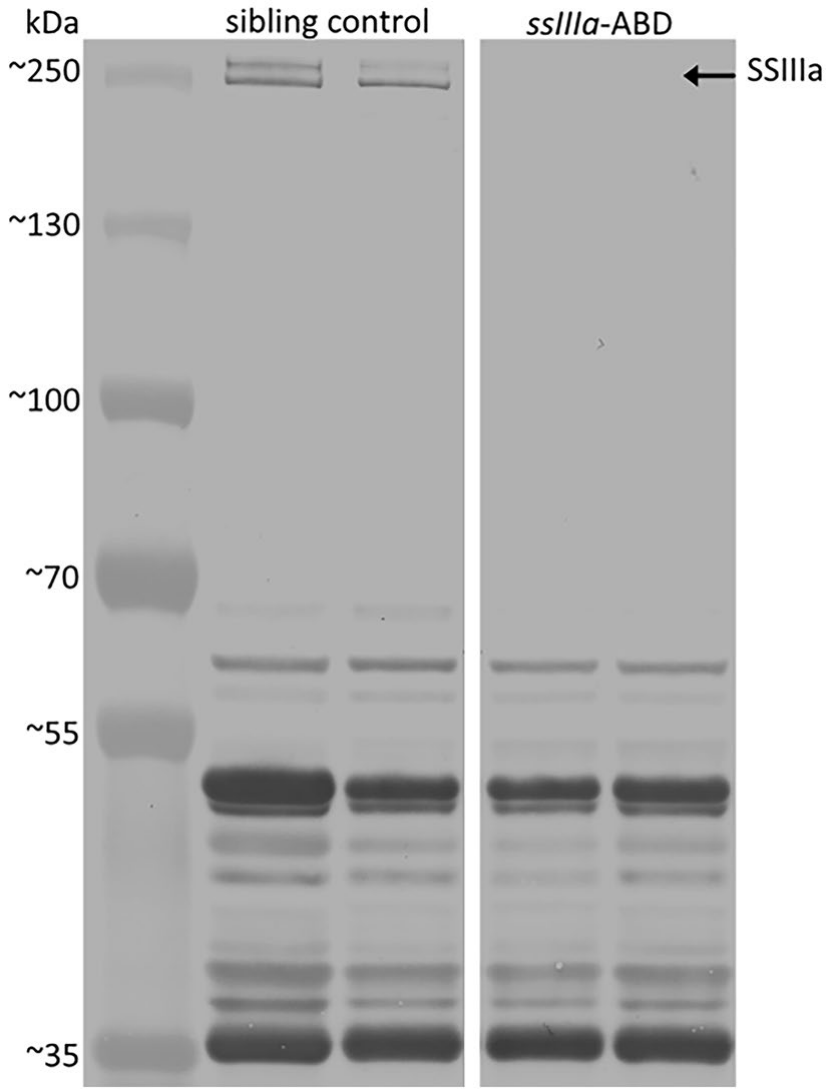
Immunoblot analysis of soluble wheat endosperm extracts separated by SDS-PAGE using maize anti-DU1N antiserum^25^. Soluble endosperm extracts of two sibling control plants and two triple mutant plants (ssIIIa-ABD) were prepared from developing grains 25 days post anthesis. Each lane contained material from 0.2 mg fresh weight of endosperm from separate plants. The leftmost lane was loaded with 5 µl of PageRuler Plus Prestained Protein Ladder (Thermo Scientific). The masses of the marker proteins in kDa are indicated. Different parts of the same blot were used to make this figure; the white space between the blot sections indicates where the image was cropped. The uncropped original blot image is in the Supplementary Material and also includes additional samples from other plants (Figure S3).

To determine the effect of *ssIIIa* mutations on SSIIIa protein, immunoblot analysis was performed using maize polyclonal antiserum for SSIIIa (anti-DU1N)^25^ (Fig. 1). The anti-DU1N antiserum recognized multiple proteins in extracts of sibling control grains. However, all the bands except two bands at ~ 250 kDa and one at ~ 70 kDa were also present on blots of the triple mutants, thus the common bands were presumed to result from non-specific binding of the antiserum and/or recognition of other wheat starch synthase isoforms. The predicted molecular weights of the mature SSIIIa proteins corresponding to the A, B and D homoeologues were calculated as 183 kDa, 181 kDa and 181 kDa respectively, which is consistent with the 183 kDa molecular weight predicted by Li et al.^8^. Wheat, barley, maize and rice SSIII proteins were previously reported to have anomalously low electrophoretic mobilities; it has been suggested previously that this could be due to intrinsic properties or post-translational modifications^8,21,23,25^. For example, the SSIIIa protein of maize is predicted to encode a 188-kDa protein; however, in SDS-PAGE of grain extracts the protein migrated with an apparent mass in excess of 200 kDa^25^. We conclude that the bands at 250 kDa are highly likely to be SSIIIa. Their absence in the triple mutant is consistent with loss of all three homoeologous proteins.

## Grain characteristics

### Grain physical characteristics

**Figure 2:**
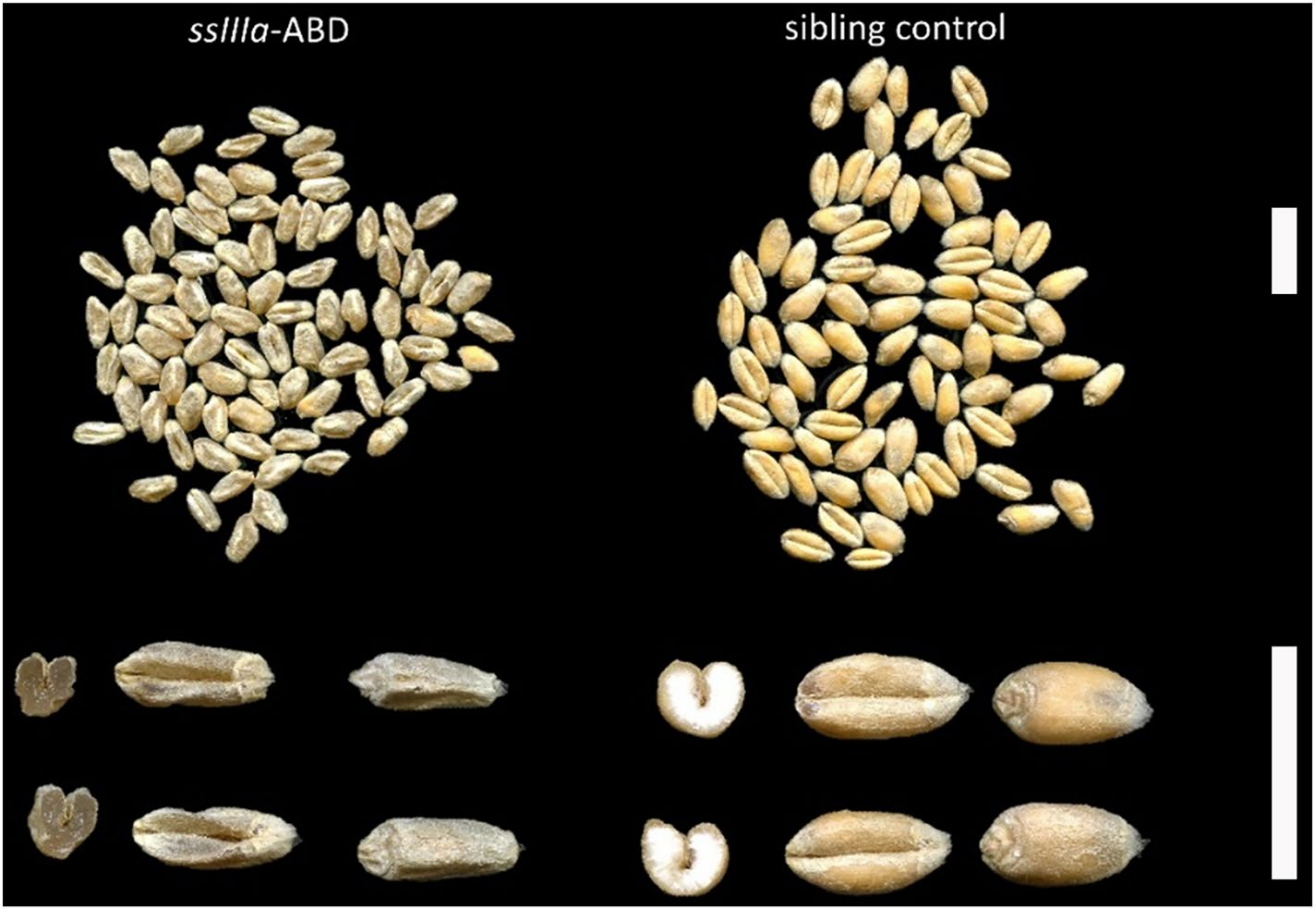
Whole grains and cross-sections of the *ssIIIa*-ABD triple mutant (left) and sibling control (right). Size bars represent 1 cm.

At maturity, most of the triple mutant grains were shrunken and dull in colour compared to plump and yellow sibling control grains (Fig. 2). Further examination by dissection showed vitreous endosperms in triple mutant grains and white mealy endosperms in sibling control grains (Fig. 2). The grains of single and double mutants were not noticeably different from sibling control grains.

The average thousand grain weight (TGW) of triple mutants was 11% lower than that of sibling controls (37.7 g vs. 42.3 g for sibling controls, *P* = 0.004); the average width of triple mutant grains was also 5% lower than that of the sibling control (*P* < 0.001) and the area was 5% smaller (*P* = 0.04) (Tables 2 and S4). The grains of single and double mutants were not significantly different from the sibling control with respect to these physical characteristics.

**Table 2:** TGW, protein and starch properties of mature grains of *ssIIIa* mutants and sibling control.

	TGW (g)	Protein (%)	Starch		Amylose		Resistant starch	
			g/100 g wholemeal flour	mg/grain	% (iodine method)	% (SEC method)	g/100 g wholemeal flour	g/100 g starch
Sibling control	42.3 (2.8)	9.1 (0.5)	68.1 (5.6)	28.7 (1.1)	26.0 (3.2)	25.5 (1.7)	2.86 (1.15)	0.69 (0.11)
*ssIIIa*-A	41.3 (3.0)	9.4 (0.9)	65.8 (4.7)	27.0 (3.2)	26.7 (1.8)	25.3 (1.4)	2.40 (0.72)	0.72 (0.16)
*ssIIIa*-B	43.5 (3.4)	8.0 (1.6)	64.4 (2.5)	27.9 (3.0)	27.3 (1.3)	27.0 (2.4)	2.59 (0.89)	0.80 (0.13)
*ssIIIa*-D	40.8 (4.2)	8.1 (2.1)	64.4 (2.1)	27.2 (2.6)	25.5 (2.8)	27.5 (3.0)	2.59 (1.28)	0.47 (0.09)
*ssIIIa*-AB	41.8 (2.0)	8.6 (0.5)	63.7 (2.5)*	26.6 (2.4)	28.9 (4.7)	29.2 (1.2)*	3.76 (0.50)	0.87 (0.20)
*ssIIIa*-AD	43.7 (4.5)	9.2 (2.0)	61.4 (3.0)*	26.9 (3.9)	26.4 (3.1)	28.9 (1.0)*	3.75 (0.50)	0.78 (0.27)
*ssIIIa*-BD	39.4 (3.5)	8.9 (1.7)	59 (2.9)*	23.3 (2.3)*	26.3 (2.4)	29.9 (3.0)*	3.98 (1.65)	0.69 (0.06)
*ssIIIa*-ABD	37.7 (3.0)*	10.3 (2.1)	56.1 (2.7)*	20.3 (2.2)*	35.1 (0.56)*	33.8 (4.0)***	6.01 (0.61) ***	1.70 (0.59)***

Protein, starch and resistant starch contents of wholemeal flour were measured on a fresh weight basis. Starch content (mg/grain) was calculated using TGW values. Values represent the mean and (standard deviation) for n = 5 biological replicates (individual plants), except for TGW and protein which is for 5 ≥ n ≤ 13 biological replicates (sample sizes in Table S5). Statistically significant differences (*P* < 0.05) identified in regression models comparing mutant genotypes to the sibling control are indicated with an asterisk (*).

### Hardness and germination potential

Given the vitreous nature of triple mutant grains, we compared the hardness index and germination potential of these grains with those of the sibling controls. Triple mutant grains were 32% harder than sibling control grains, 79.0 (SD = 4.2) vs. 59.6 (SD = 9.7) for controls, (*P* = 0.003, Table S4). The hardness of a sample is reported in arbitrary units and values above 45 are termed hard; typical bread wheat ranges from 50 to 80^30^. The vitreous nature of the triple mutant grains had little effect on their germination potential: the germination index was only 6% lower than that of sibling controls (*P* = 0.041, Table S4).

### Grain protein content

There was no evidence for an effect of genotype on mature grain protein content (estimated by measuring nitrogen released by combustion) (e.g., triple mutant = 11.27% (SD = 2.04%), compared to 9.91% (SD = 0.75%) in the sibling control; ANOVA across all genotypes *P* = 0.400) (Table S4).

## Starch properties

### Starch content

Wholemeal flour prepared from triple mutant grains had significantly less starch than the sibling controls (56.1 g/100 g flour compared to 68.1 g/100 g flour in sibling controls; *P* < 0.001) (Table 2, Fig. S4a). The flour starch contents of the double mutants were also significantly lower than the sibling control (Table 2, Fig. S4a). Although for the single mutants, there were no significant differences compared to the sibling control, the values were lower suggesting a possible dose–response effect of number of mutations on starch content (Table 2, Fig. S4a). The flour starch content was used to calculate the starch content on a per grain basis using thousand grain weight (TGW) values. Starch contents per grain for the triple mutants were significantly lower than for the sibling controls (20.3 mg/grain compared to 28.7 mg/grain in sibling controls; *P* < 0.001), as were the *ssIIIa*-BD double mutants (23.3 mg/grain; *P* = 0.003 compared to sibling controls) (Table 2). Similarly, the starch contents per grain for each of the single and the other double mutants were lower albeit not individually statistically significantly different from sibling controls (Table 2).

### Amylose and resistant starch

The amylose content of purified starch from triple mutant grains measured by iodine binding was higher than that of the sibling controls (35.1% vs. 26.0% in sibling controls; *P* < 0.001), but the amylose contents of starch from single and double mutants were not different from that of the sibling controls (Table 2, Fig. S4b).

The resistant starch content of the triple mutant wholemeal flour (6.01 g/100 g flour) was more than twice that of the sibling control (2.86 g/100 g flour); *P* < 0.001) (Table 2, Fig. S4c). Resistant starch contents were also measured in purified starch samples and similarly the triple mutants (1.70 g/100 g starch) had nearly 2.5-fold more than the sibling controls (0.69 g/100 g starch; *P* < 0.001) (Table 2).

### Starch granule morphology and size distribution

**Figure 3:**
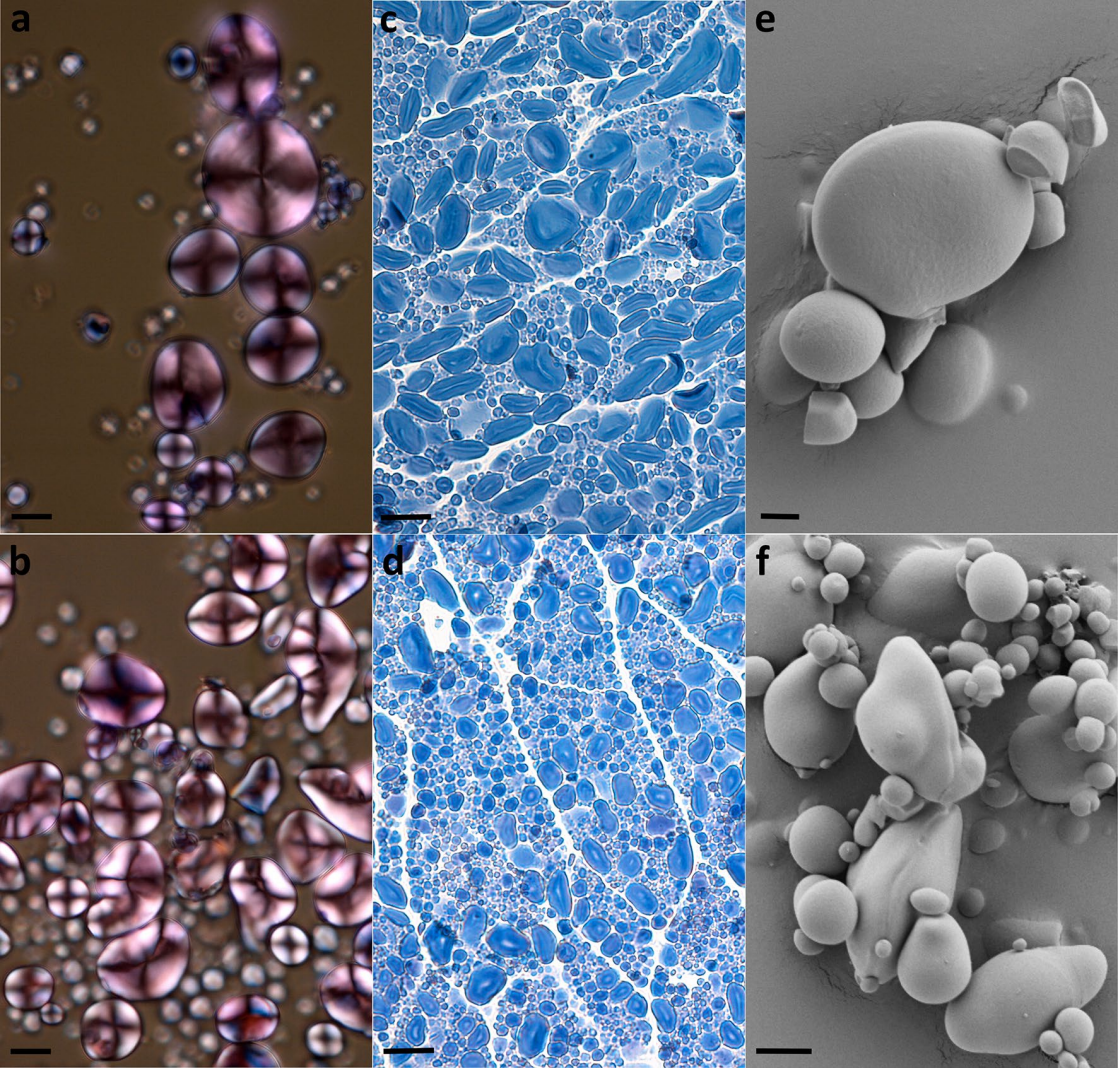
Light and scanning electron microscopy of the sibling control (top panels: **a**,**c,e**) and triple *ssIIIa* mutant (bottom panels: **b**,**d**,**f**). Panels (**a**,**b**) are images of isolated starch stained with Lugol’s solution and viewed with a polarizing filter; size bars represent 10 µm. Panels (**c**,**d**) are images of grain sections stained with Lugol’s solution; size bars represent 25 µm. Panels (**e**,**f**) are images of isolated starch; size bars represent 2 µm for panel (**e**) and 5 µm for panel (**f**).

Light microscopy was used to examine isolated starch and grain sections of the *ssIIIa* mutants and sibling controls. In the triple mutants many of the A-type granules were smaller and more elongated than those of sibling controls, and many granules had protrusions (Fig. 3). These characteristics were also observed using scanning electron microscopy of isolated starch (Fig. 3). The birefringence pattern of many of the triple mutant A-type granules in polarized light showed distorted cross patterns distinct from the typical ‘Maltese cross’ pattern observed in the sibling control; this was more apparent in granules that had an irregular shape and protrusions. The birefringence pattern of the B-type granules was not notably different (Fig. 3).

**Figure 4:**
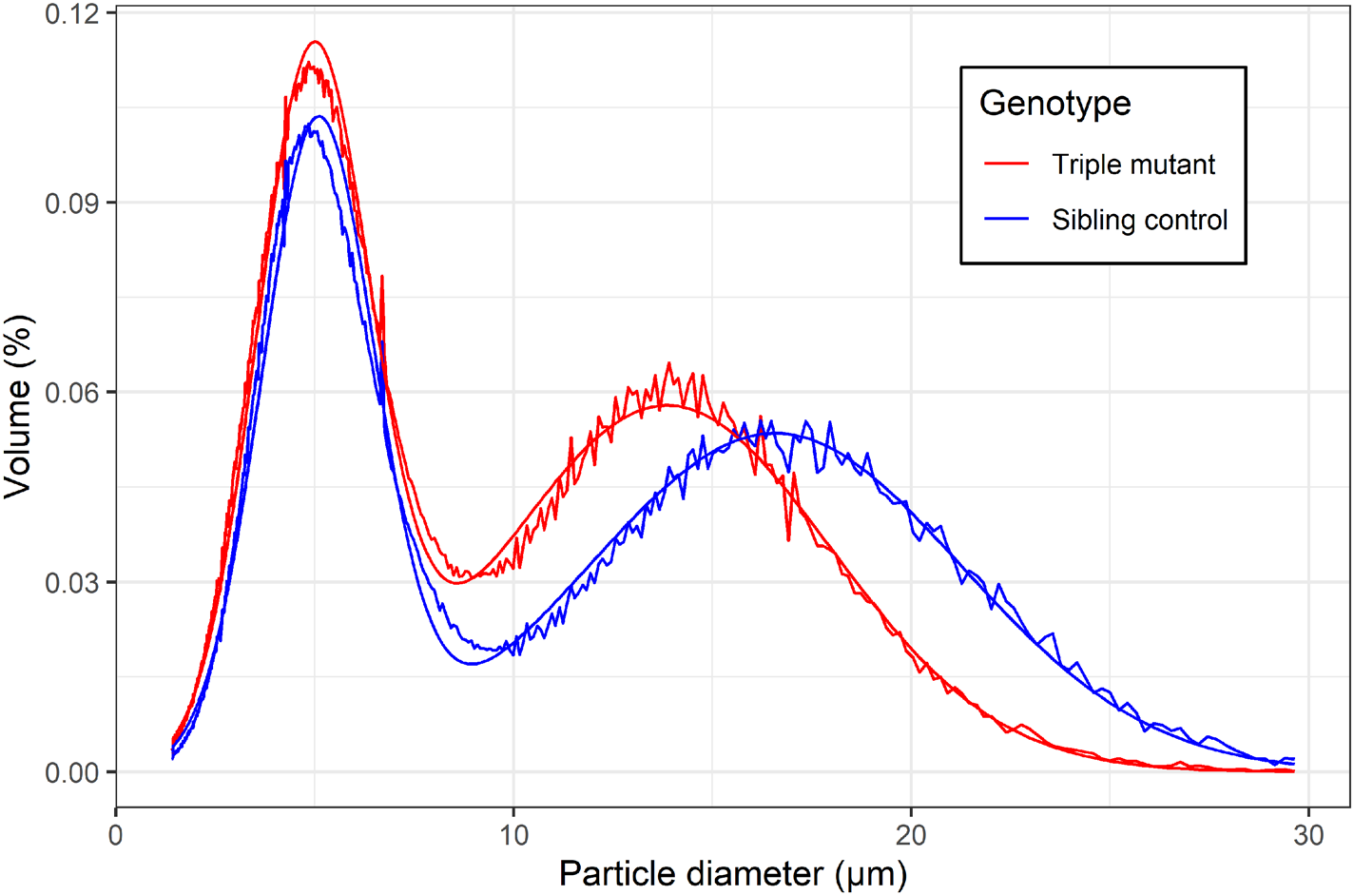
Starch granule size distribution of triple mutants (*ssIIIa*-ABD) and sibling controls. Volume values represent the average observed proportion of volume accounted for by each particle diameter for n = 5 biological replicates per genotype, with average two-component normal mixture models for both genotypes overlaid.

To quantify starch granule size and size distribution a Coulter counter (Beckman Multisizer 4e) was used to measure particle volumes in purified starch. All of the starch samples showed a bimodal size distribution as expected for wheat^31^, and these were estimated as mixtures of two Gaussian distributions individually for each replicate. There was a clear shift towards smaller A-granule diameters (between 10 and 20 μm) in the triple mutant compared to the sibling control (Fig. 4).

The average size of the A-type granules was significantly smaller in the triple mutant (13.9 µm, SD = 0.75; *P* < 0.001) and BD double mutant (15.6 µm, SD = 0.71; *P* = 0.019) compared to sibling controls (16.7 µm, SD = 0.28) (Table S4). There was no significant difference in the proportion of the larger A-type granules across genotypes, or in the diameter of the B-type granules (Table S4).

### Starch chain-length distribution

**Figure 5:**
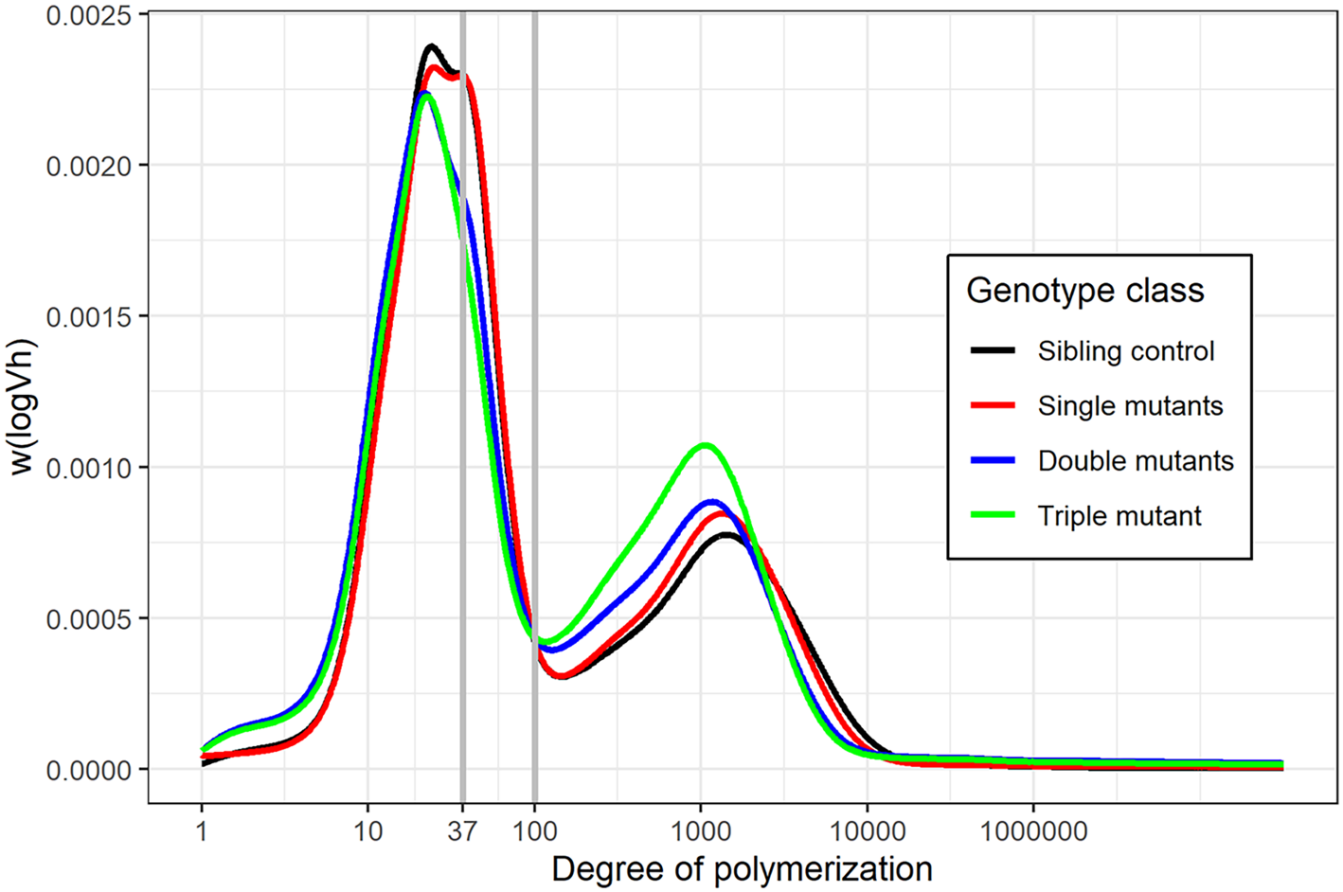
Average chain-length distributions of debranched starch. The y-axis, W(logVh) represents the weight distribution based on the relationship between elution volume and hydrodynamic radius (logVh) for pullulan standards, scaled so that the area under each curve is constant. Each line represents the average for each genotypic class: wild-type sibling control (n = 5), single *ssIIIa* mutants (n = 15), double *ssIIIa* mutants (n = 15) and triple *ssIIIa* mutant (n = 4).

Both double and triple *ssIIIa* mutants had different chain-length distributions (CLD) compared to the sibling control (measured on debranched starch). Analysis of CLD data indicated fewer long amylopectin chains and more amylose chains in the double and triple mutants compared to the sibling controls. The CLDs showed three distinct peaks, the first around a degree of polymerization (dp) of 10–12 corresponding to short amylopectin chains within a single crystalline lamella of starch, the second between 37 and 100 dp corresponding to longer chains that span multiple lamellae as defined by Hanashiro, et al.^32^, and the third around 2500 dp corresponding to long amylose chains^32^ (Fig. 5).

The relative heights of the first and third peak, and the proportion of areas under the curve in regions corresponding to < 37 dp, 37–100 dp and > 100 dp were calculated for starch from individual plants (biological replicates, Table S5) for each genotype (wild-type sibling control, single: *ssIIIa*-A, *ssIIIa*-B, *ssIIIa*-D; double: *ssIIIa*-AB, *ssIIIa*-AD, *ssIIIa*-BD; triple: *ssIIIa*-ABD). The relative heights of the peaks were significantly different between genotypes (1-way ANOVA, *P* < 0.001). The proportion of area > 100 dp (corresponding to proportion amylose by mass) was higher in the double (mean = 29.4%) and triple mutants (mean = 33.8%) than the sibling control (mean = 25.5%; *P*-values for differences = 0.003 and < 0.001, respectively). Within chains < 100 dp; the proportion of the area corresponding to longer chains (37 to 100 dp) was lower in the double (30.0%) and triple mutants (28.3%) than the sibling control (35.5%; *P* < 0.001 for both comparisons), indicating a reduction in longer amylopectin chains compared to shorter amylopectin chains in the double and triple mutants (Table S4).

### Physicochemical properties

The elevated levels of resistant starch in the triple mutant suggest it may be useful for wheat breeding applications, thus, we examined whether the physicochemical properties of starch from the triple mutant were altered relative to those of the controls. The gelatinization behaviour of purified starch was measured using differential scanning calorimetry (DSC). The peak and conclusion temperatures of gelatinization for the triple mutants were significantly greater than those of the sibling controls (for peak: 61.8 °C vs. 58.4 °C in sibling controls (*P* < 0.001); for conclusion: 75.4 °C vs. 70.5 °C in sibling controls (*P* = 0.001)) (Table S4). There were no significant differences in enthalpy and onset temperature between the triple mutants and sibling controls (Table S4).

### Soluble sugars

Soluble sugars (glucose, fructose and sucrose) were measured in grains of triple mutant and wild-type sibling controls harvested at 25 days after anthesis (DAA) and maturity. At 25 DAA, sucrose levels in the triple mutant were significantly greater, 3.04 mg/grain vs 1.00 mg/grain in the sibling control, (*P* < 0.001) (Table S6). At maturity, the triple mutant had both higher levels of fructose (0.18 mg/grain vs 0.10 mg/grain in the sibling control, *P* = 0.042) and sucrose (1.09 mg/grain vs 0.57 mg/grain in the sibling control, *P* = 0.0037) (Table S6). Overall, the total level of soluble sugars (sum of glucose, fructose and sucrose) at 25 DAA and maturity were higher in the triple mutant compared to the wild-type sibling controls (*P* = 0.0001 and *P* = 0.0005 respectively) (Table S6).

### Non-starch polysaccharides

To determine pleiotropic effects of *ssIIIa* mutations on non-starch polysaccharides in the grain, enzyme fingerprinting was used to measure relative levels of arabinoxylan oligosaccharides (AXOS) and mixed-linked β-glucan oligosaccharides in wholemeal flour samples following enzymatic digestion. Total AXOS (sum of peak areas of all known AXOS peaks) was significantly higher in the triple mutants (35% more, *P* < 0.001) and in BD double mutants (17% more *P* = 0.001) relative to the wild-type sibling controls (Table 3), with some evidence for a dose–response effect among single mutants and the AB and AD double mutants. Both the triple and BD-double mutants also had greater levels of substituted and unsubstituted AXOS compared to the wild-type siblings. Interestingly, the other double mutants (AB and AD) and D-genome single mutants showed greater levels of substituted AXOS, reflected in the small change in total AXOS (Table 3). Only the AD-double mutants had a greater ratio of substituted:unsubstituted AXOS compared to the wild-type sibling controls (0.67 for AD, and 0.56 for sibling controls, *P* = 0.008) (Table S4).

**Table 3:** Arabinoxylan oligosaccharides (AXOS) and mixed-linkage β-glucans (MLGs) in wholemeal flour of *ssIIIa* mutants and sibling control.

	Relative peak values			
	Total AXOS	Substituted AXOS	Unsubstituted AXOS	MLG (G3 + G4)
Sibling control	32.7 (3.6)	11.6 (0.4)	21.0 (3.3)	9.1 (1.2)
*ssIIIa*-A	32.8 (2.4)	11.7 (0.3)	21.0 (2.6)	9.3 (1.1)
*ssIIIa*-B	34.1 (2.3)	12.0 (0.9)	22.1 (1.7)	9.7 (0.6)
*ssIIIa*-D	34.0 (1.9)	12.9 (0.4)*	21.1 (1.7)	10.8 (0.4)*
*ssIIIa*-AB	35.3 (2.7)	13.2 (1.3)**	22.1 (1.5)	10.6 (0.6)*
*ssIIIa*-AD	35.1 (2.9)	14.0 (1.1)***	21.2 (2.3)	10.0 (1.4)
*ssIIIa*-BD	38.2 (3.2)**	14.3 (0.7)***	24.0 (2.6)*	10.6 (0.6)*
*ssIIIa*-ABD	44.1 (1.9)***	16.7 (0.8)***	27.4 (1.6)***	12.7 (1.5) ***

Values were calculated as the sum of peaks relative to the internal standard and represent the mean and (standard deviation) for n = 5 biological replicates. MLGs were calculated by summing G3 + G4, the two major β-glucan oligosaccharides released by enzymatic digestion. Significant P-values (P < 0.05) identified in regression models comparing mutant genotypes to the sibling control are indicated with an asterisk (*).

We also observed higher levels of mixed-linkage β-glucans (MLGs) in the triple mutant (40% more, *P* < 0.001), AB and BD double mutants (both 17% more, *P* = 0.016 and *P* = 0.017 respectively), and D-genome single mutant (19% more, *P* = 0.009), with some evidence for an increase in the B and AD mutants (Table 3). There were no differences in the G3 to G4 ratio for each genotype compared to the wild-type sib suggesting no changes in the structure of MLGs (Table S4).

## Discussion

The aim of our study was to estimate the nature and extent of alterations in starch and grain properties brought about by loss of SSIIIa in wheat. Research in other cereal crops has shown major effects of loss of SSIII that include increased resistant starch content^20–22^. Our data suggests that the loss of function of all three *ssIIIa* homoeologues produces substantial effects on starch properties and grain physical characteristics, and that the effect of double and triple mutation tends to be greater than the added effects of single mutations. The phenotypic effects observed in double mutants were unanticipated given that suppression of all homoeologous copies of a gene in wheat is often required to influence phenotype^33^. For example, single and double *sbeIIa* hexaploid wheat TILLING mutants showed no significant differences in amylose content compared to wild-type siblings, but lines with mutations in all three *sbeIIa* homoeologues had 55.7% amylose compared to 22.9% measured in the wild-type sibling control^13^. In contrast, some of our results, namely starch chain-length distribution, clearly indicate that suppression of two homoeologous *SSIIIa* copies is enough to significantly affect phenotype. Similarly, a report of *ssIIa* single-homoeologue mutants indicated various effects on starch composition and another study investigating *waxy* (*gbssI*) mutants presented differential effects of distinct Waxy proteins (Wx-A1, -B1 and D1) on amylose content^34,35^. These findings suggest that starch gene homoeologues do not always show functional redundancy but can also present dosage and homoeologue-specific effects. This presents new opportunities for fine-tuning starch phenotypes in breeding applications to reach desired effects on starch structure which is not possible in diploid crops like rice or maize.

The triple *ssIIIa* mutants differed strongly from controls with respect to starch granule morphology and size distribution. Wheat has two distinct types of starch granules, disc-shaped A-type granules of 5–40 μm in diameter and more spherical B-type granules which are smaller (< 5 μm) and more abundant^36^. The smaller A-type granules observed in the triple mutant suggest that SSIIIa plays a major role in the growth of A-type granules. Many of the A-type granules also had an unusual shape; this could be due to an effect on granule growth and/or the changes in starch composition (more amylose) and amylopectin structure (fewer long chains). The change in birefringence pattern observed in the A-type granules of triple mutants is also consistent with irregular patterns of granule growth. In contrast, the B-type granules in the triple mutant appeared normal which could suggest a more specific role of SSIIIa in the synthesis of A-type granules. Peng, et al.^37^ previously showed preferential binding of starch branching enzyme isoforms to A-type granules and proposed the proteins could play a role in the growth of small A-type granules into full-sized A-type granules; it is possible that SSIIIa could play a similar role. We also observed A-type granules with a more normal shape; the mixture of normal and abnormal granules may reflect an increasing influence of SSIIIa on granule growth as cellularisation and granule initiation proceeds during the very early stages of endosperm development. Initiation of A-type granules begins very early in development when *SSIIIa* expression is relatively low and continues for a few days as SSIIIa transcript levels rise and *SSIIIb* transcript levels fall. It is thus possible that the granules formed early in development in the triple mutant have fewer growth defects than those formed towards the end of the A-type granule initiation period^38^.

Disruption of granule size and morphology due to loss of SSIIIa has been observed in other cereals. Wang, et al.^39^ previously reported that *du1* mutants in maize had smaller starch granule size distributions (4–11 μm) than a control (6–17 μm). In rice, the starch granules of a *ssIIIa* mutant were variable in size and more rounded in shape than normal polyhedral starch granules^22,40^. Ahmed, et al.^41^ reported A-granules with ‘bumps’ in barley *amo1* mutants like the protrusions we observed in the wheat triple *ssIIIa* mutants. It is worth noting that starch granule size distribution can affect physicochemical properties of flour and breadmaking properties, which may be an important consideration for possible applications of the *ssIIIa* mutant^42^.

The amylose content of the starch of the triple *ssIIIa* mutant had a relative increase of ~ 35% compared to the sibling control (measured by iodine binding method) (Table 2). Elevated amylose contents have also been observed in other cereals carrying *ssIIIa* mutations; a mutant for the barley *amo1* locus which is suggested to encode *ssIIIa* had ~ 40% more amylose than a wild-type control and a rice *ssIIIa* mutant had ~ 33% more amylose relative to the wild-type parent^21,23^.

The starch chain-length distribution profiles of the double and triple *ssIIIa* mutants were different from that of the sibling control (Fig. 5). Both iodine binding and SEC revealed substantial elevations of amylose in the triple mutant, and SEC also revealed smaller but significant elevation in the double mutants (iodine binding: 35.1% for triple mutants vs. 26.0% in sibling controls; SEC: 33.8% for triple mutants and 29.3% for double mutants vs. 25.5% in sibling controls). The different levels of amylose observed in double vs. triple mutants may be of particular interest for wheat breeding applications and open new possibilities for fine-tuning amylose levels in wheat using unique combinations of mutant *ssIIIa* homoeologues. The double and triple mutants also had fewer long amylopectin chains; this was anticipated given the proposed role of SSIII in producing longer length amylopectin chains by extending short chains produced by SSI^43^. Prior studies of *ssIIIa* mutants in maize and rice also found that the mutants had fewer long amylopectin chains^20,23^. Given that the starch content was lower in the flour of double and triple mutants, it seems likely that the higher amylose contents are almost entirely due to the reduction in the rate of synthesis of amylopectin, rather than an increase in the absolute rate of amylose synthesis (Table 2). For instance, based on starch content of wholemeal flour and percent amylose measured by SEC reported in Table 2, the triple mutant contained 19 g amylose per 100 g wholemeal flour, and the control contained 17.4 g amylose per 100 g flour; thus there is less than a 10% difference in the amylose content of triple mutant and control flour.

A key aim of our study was to discover if loss of SSIIIa impacts levels of resistant starch in wheat flour and purified starch to determine the potential value of the *ssIIIa* mutations for wheat breeding. Wholemeal flour prepared from *ssIIIa* triple mutant grains had about twice the amount of resistant starch than flour from the sibling controls (6.01 g/100 g of flour vs. 2.86 g/100 g of flour in sibling controls). It is likely that this increase is mainly due to the elevated proportion of amylose, which is less readily digested than amylopectin. The amylose content of starch typically has a positive association with resistant starch^9^. This elevation of resistant starch in the triple mutant is within the range of values reported for other starch biosynthesis mutants in wheat. For example, *ssIIa* and *sbeIIa* triple mutants produced using the cultivar Cadenza have 1.4% and 7.2% resistant starch respectively. However the amylose levels of those mutants (45.7% for *ssIIa* and 78.7% for s*beIIa*) are greater than the *ssIIIa* triple mutant (35.1%), which suggests other factors besides amylose are likely contributing to the change in resistant starch such as changes in amylopectin structure and possibly interactions with other components of the flour^17^. The lower resistant starch levels observed in purified starch compared to wholemeal flour (1.70 g/100 g of starch vs 6.01 g/100 g of flour in the triple *ssIIIa* mutant) also highlights the importance of flour matrix. Furthermore, in this study we found that mutations in *ssIIIa* genes also led to greater levels of arabinoxylans and β-glucans (Table 3), non-starch polysaccharides derived from cell walls which are the main source of dietary fibre in wheat flour^3^. It is possible that the reduction in starch biosynthesis in *ssIIIa* mutants led to a redirection of substrates to other non-starch biosynthetic pathways, a mechanism previously suggested to explain elevated levels of non-starch carbohydrates in *ssIIa* wheat mutants^16,17^. For future studies, it will be important to investigate the other complex factors that can affect levels of resistant starch including associations between starch and flour components like proteins or fibres and effects of processing methods like baking^9^.

The greater peak and conclusion temperatures observed in DSC analyses suggest that the triple mutant starch behaves differently from the sibling control when subjected to hydrothermal treatment which is consistent with previous literature for starches from a range of botanical origins with elevated amylose contents^44–46^. It will be important to discover how starch from the triple mutant behaves during processing as retrogradation can have significant effects on starch digestibility^47^. For example, retrogradation of starch in durum wheat semolina led to elevated levels of resistant starch compared to raw unprocessed semolina^4^. Similarly, the levels of resistant starch in bread crumb (2.1 g/100 g) were higher than those in raw flour (0.4 g/100 g) used to bake bread^19^.

Importantly, our results show that lack of SSIIIa has effects on the grain beyond starch composition and structure that will need to be taken into consideration for breeding applications. The grains of the triple mutants weighed about 11% less than the grains of the sibling control (Fig. 3 and Tables 2 and S4). The reduction in starch content per grain was greater than the reduction in weight of individual grains (starch was 67.8% of grain weight in the control but only 53.8% in the triple mutant) hence levels of other grain constituents (e.g., non-starch polysaccharides) accounted for more of the grain weight in the triple mutant than in control grains. Furthermore, the shrunken nature of the triple mutant grains suggested potential increases in soluble sugars, so we also measured glucose, fructose and sucrose contents of the grains at 25 days DAA prior to water loss and at maturity (Table S6). Total sugar (sucrose, glucose and fructose) per grain at 25 DAA was over two fold higher in the triple mutant (3.8 mg/grain) than the control (1.6 mg/grain), consistent with the idea that the reduction in starch accumulation is accompanied by higher sugar levels, leading to greater water uptake and hence shrinkage during grain maturation (Table S6). At maturity both the triple mutant and control had less sugar than grains harvested at 25 DAA, and the sugar content of triple mutants remained significantly greater than the controls (1.36 mg/grain vs 0.74 mg/grain in the controls).

The *ssIIIa* triple mutants were also very different from controls with respect to grain texture: mutant grains were harder and had vitreous endosperms. This is an important consideration in assessing the value of the triple mutants for wheat breeding as their hardness index was at the high end of the range considered acceptable for bread wheats; hard grains impact important milling and baking quality factors like milling energy, starch damage and water absorption^48^. Variation in grain texture of wheat can be caused by several genetic and environmental factors, including the nature and levels of puroindolines (proteins that bind to starch in flour), total protein levels and levels of water-soluble pentosans and lipids^49^. The two starch synthesis mutants of hexaploid wheat described previously—*ssIIa* and *sbeIIa*—both have hard endosperms associated with low starch contents and elevated levels of protein and water-soluble pentosans^17,19^. It seems likely that the hardness and vitreosity of *SSIIIa* grains are attributable to compositional changes other than protein since the levels of protein were not found to differ.

## Conclusions

In this study we show that lack of SSIIIa in hexaploid wheat has significant effects on starch and grain properties. Our analyses revealed interesting gene dosage effects which could be used for fine-tuning starch properties in wheat breeding applications. Although amylose and RS were elevated in mutants, the altered grain characteristics, may limit their usefulness for wheat breeding. Reductions in starch content and grain weight are a common effect of starch biosynthesis mutations and are likely to lead to yield penalties^12,18,19^. Further studies using backcrossed and independent triple mutant lines and evaluation of field performance and end-use functionality will shed light on whether the *ssIIIa* mutant offers any advantages for commercial breeding.

## Materials and methods

All methods were carried out in accordance with relevant guidelines and regulation.

### Plant materials

For each *SSIIIa* homoeologue (A, B and D), TILLING mutants carrying premature stop mutations were identified in the hexaploid wheat variety Cadenza using the in silico Wheat TILLING database (http://www.wheat-tilling.com/)^29^. Grains for each mutant were ordered from the Germplasm Resources Unit (John Innes Centre in Norwich, UK) using the publicly accessible SeedStor system https://www.seedstor.ac.uk/; permission to use the materials for research purposes was obtained. The mutants were crossed, and the single, double, and triple mutants were selected from segregating F_2_ populations along with sibling controls not carrying the mutations in *SSIIIa*. Selections were made using KASP genotyping (LGC Biosearch Technologies, https://www.biosearchtech.com/) with homoeologue specific primers designed using the program PolyMarker^50^. Mutations were validated in triple mutants by Sanger sequencing; the sequencing primers are available in Table S3.

Selected F_2_ plants were grown in the glasshouse in a randomized block design and grains harvested at maturity. To make wholemeal flour, 5 grain samples, each from a different plant, were selected at random and ground in a UDY Cyclone Mill (UDY Corporation, https://www.udyone.com/) using a 0.5 mm screen. The same grain samples were used for starch purification. For each outcome measured, biological replicates are defined as samples originating from separate F_2_ plants. (Sample sizes are reported in Table S5).

### Immunoblot analysis

Developing grains at 25 days after anthesis were harvested from F_2_ plants into liquid nitrogen. Frozen grains were homogenized in SDS-PAGE loading buffer and centrifuged at ~ 20,800*g* for 20 min. The supernatant was heated for 10 min at 70 °C and centrifuged again for 5 min. The supernatant was separated by SDS-PAGE on an 8% polyacrylamide gel. The gel was electroblotted onto PVDF membrane and developed with primary antibody: rabbit serum containing antibodies against the N-terminal region of the DU1 (SSIII) protein, then a goat anti-Rabbit IgG-Alkaline Phosphatase antibody. Blots were developed with BCIP/NBT Colour Development Substrate (5-bromo-4-chloro-3-indolyl-phosphate/nitro blue tetrazolium) following manufacturer instructions (Promega S3771).

### Grain properties

Thousand grain weight, grain length, grain width and grain area were estimated from the weight of 387–1171 mature grains per plant using a MARVIN seed analyzer (MARViTECH, https://www.marvitech.de). The protein content of mature grains was measured using the AACC 46–30 Crude protein—combustion method^51^ on a CE440 Elemental Analyser (Exeter Analytical, https://www.exeteranalytical.co.uk/). Grain hardness index was determined using a Perten Single Grain Characterization System (PerkinElmer, https://www.perkinelmer.com/uk); the average of 100 grains was measured for each biological replicate.

### Germination index

For each sample (biological replicate) 50 grains were surface sterilized and incubated at 17 °C on filter paper in the dark. The germination index was calculated using the method of Walker‐Simmons^52^.

### Starch properties

#### Isolation of starch from mature wheat grains

For each sample approximately 50 wheat grains were soaked overnight then homogenized in a small volume of water using a mortar and pestle. The homogenate was filtered through Miracloth and the filtrate centrifuged at 3000*g* for 5 min. The pellet was washed twice with 2% (w/v) SDS then twice with ice cold acetone by resuspension followed by centrifugation. The final pellet was air-dried.

#### Total starch and resistant starch

Total starch content was measured for 100 mg samples of wholemeal flour using the Total Starch Assay kit (Megazyme International Ireland Ltd. Co., https://www.megazyme.com/) following the manufacturer’s instructions for the recommended KOH assay format. The resistant starch content (amount of resistant starch measured on a g/100 g ‘as is’ basis) was measured for 100 mg samples of wholemeal flour and 100 mg samples of purified starch using the Resistant Starch Assay Kit (Rapid) (Megazyme International Ireland Ltd. Co., https://www.megazyme.com/).

#### Amylose

The apparent amylose content was determined using an iodine dye binding method using a potato amylose standard curve^53,54^. Apparent amylose contents were calculated from the amylose standards, and these were converted to true values using the following equation: % amylose = % apparent amylose—(6.2/93.8), which corrects for absorbance from amylopectin binding iodine^53^.

#### Starch chain-length distribution

Purified starch isolated from whole grains was solubilized in DMSO containing 0.5% (w/v) LiBr followed by enzymatic debranching with isoamylase^55^. Starch chain-length distributions were analyzed according to the HPLC-SEC procedures detailed in Supplementary Material. Three parameters were estimated for each genotype; these were (1) the relative heights of the peaks corresponding to long amylopectin chains and amylose, (2) the ratio of the area under the curve > 100 dp (amylose) to total area, and (3) the ratio of the area between 37 and 100 dp (long amylopectin chains) to the area between 0 and 100 dp (total amylopectin) as defined by Hanashiro, et al.^32^. Since each outcome was relative within the sample, no cross-sample normalisation was necessary. These ratios were then compared as other outcomes as described in the Data analysis section below.

#### Differential scanning calorimetry

Differential scanning calorimetry was used to characterize the thermal properties of starch using a MCDSC instrument, TA Instruments (Elstree, UK). Starch (50 mg) was accurately weighed in a high-pressure pan (1 mL capacity, Hallestoy) then 1 mL of degassed water added and the pan lid hermetically sealed. The furnace was purged with dry nitrogen gas at a flow rate of 50 mL/min. The measurement procedure started with temperature equilibration at 20 °C for one hour, followed by a temperature ramp up to 140 °C at the rate of 1 °C/min. DSC parameters were analysed using TA NanoAnalyze and TA Advantage/Universal Analysis.

#### Starch granule size distribution

Starch granule size distribution was measured using a Coulter counter (Beckman Coulter Multisizer 4e). The average of two technical replicates was used for each biological replicate. The relative total volume of A-type and B-type starch granules was estimated for each technical replicate by fitting the distribution of diameters (weighted by particle volume) to a mixture of Gaussian distributions, which was observed to fit the data well in all cases. From each mixture model we extracted the relative proportion of total starch volume accounted for by B-type granules, and the mean diameters of the A-type and B-type granules. These were then compared across genotypes as other outcomes using the linear mixed models described in the Data analysis section.

### Non-starch polysaccharides

Enzyme fingerprinting was used to measure relative values of arabinoxylan oligosaccharides (AXOS), and mixed-linked β-glucan (MLG) oligosaccharides following the methods by Ordaz-Ortiz et al. (2004 and 2005)^56,57^, except that recombinant enzymes were used for digestion. Briefly, wholemeal flour samples were digested with endo 1,4 β-xylanase (E.C.3.2.1.8) (PRO-E0062, Promix Limited, UK,) and endo 1,3(4) glucanase (‘lichenase’) (E.C.3.2.1.73) (PRO-E0017, Promix Limited, UK) to digest AX and MLG, respectively. After digestion, the samples were filtered, diluted in 10 μM melibiose (internal standard), and analysed using a CarboPac PA1 analytical column (guard column: 2 × 50 mm × analytical column: 2 × 250 mm) on a Dionex ICS-3000 (Thermo Scientific). The peak areas of the oligosaccharides released by enzyme digestion were expressed as percentages of the total peak areas of all AXOS and ‘total AXOS’ was calculated as the sum of all known AXOS peaks. Two major β-glucan oligosaccharides, G3 and G4, were released by enzymatic digestion and total MLG was calculated as the sum of G3 + G4 peak areas.

### Soluble sugars

Developing grains at 25 days after anthesis and mature grains were harvested from F2 plants into liquid nitrogen. Extracts of soluble sugars were prepared and assayed enzymatically following methods reported in Fahy, et al.^58^.

### Microscopy

#### Light microscopy of isolated starch and grain sections

Two-µM sections of mature grains prepared using a microtome were placed in a drop of 50% (v/v) ethanol on a microscope slide and dried at room temperature. Grain sections and isolated starch were stained with diluted Lugol’s iodine solution (4% v/v for sections and 6.25% v/v for isolated starch) (Sigma L6146) and viewed on a light microscope. A polarizing filter was used to examine the birefringence patterns of starch granules.

#### Scanning electron microscopy (SEM) of isolated starch

Dried isolated starch was gently brushed onto a carbon tab and fixed to a SEM pin stub. Samples were viewed using a Zeiss Supra 55 VP FEG SEM.

### Data analysis

All outcomes were compared across genotypes using linear mixed effects regression models, with the sibling control chosen as the reference group. Replicates in analysis correspond to individual plants. Between 3 and 13 replicates were included for each genotype in each analysis depending on available material (see Table S5).

Where only the triple mutant and sibling control were included, a simple linear regression was used. Where outcomes were measured across all genotypes, two sets of contrasts were calculated; first each mutant class was independently compared to the sibling controls, second, the effect of each individual mutation and their two- and three-way interactions were reported. Effect sizes, standard errors and *P*-values are reported for all comparisons in Regression models in Supplementary Material. A p-value of 0.05 is adopted for statistical significance, but p-values should be read in the context of size of any difference, the ‘dose–response’ effect across genotypes and the number of independent tests that were conducted. A logarithmic transformation was used for analysis of sugars.

All analyses were conducted using R (versions 3.6.1 and 4.1.1) , using the lme4 (version 1.1-27.1) and lmerTest (version 3.1-3) packages for estimating mixed models^59–61^. The mixdist (version 0.5-5) R package was used to estimate mixture models^62^. All analysis code is available at https://github.com/quadram-institute-bioscience/SSIIIa-Rscripts.

## Supplementary Information

Supplementary Information.

## Data Availability

The datasets generated during and analysed during the current study are available in the Grassroots Data Repository at https://opendata.earlham.ac.uk/wheat/under_license/toronto/#.

## Abbreviations

AXOS: Arabinoxylan oligosaccharides
CLD: Chain-length distribution
DAA: Days after anthesis
DP: Degree of polymerization
DSC: Differential scanning calorimetry
HPLC-SEC: High performance liquid chromatography-size exclusion chromatography
MLG: Mixed-linkage β-glucan
SEM: Scanning electron microscopy
TGW: Thousand grain weight
TILLING: Targeting induced local lesions in genomes
