# Supplementary material for: Loss of starch synthase IIIa changes starch molecular structure and granule morphology in grains of hexaploid bread wheat
Source: Sci Rep. 2022 Jun 25;12:10806. doi: 10.1038/s41598-022-14995-0 (PMC9233681; doi:10.1038/s41598-022-14995-0)
Supplement: Supplementary file 1 — Supplementary Information. [file 41598_2022_14995_MOESM1_ESM.pdf]

Loss of Starch Synthase IIIa changes starch molecular structure and granule morphology in grains of hexaploid bread wheat

Brendan Fahy, Oscar Gonzalez, George M. Savva, Jennifer H. Ahn-Jarvis, Frederick J. Warren, Jack Dunn, Alison Lovegrove, Brittany A. Hazard

Supplementary Material

Table S1. *KASP primer sequences used for marker-assisted selection of ssIIIa alleles. Line is the mutant number in the TILLING population (e.g., C2074 = Cadenza 2074).*

Gene	Line	Primer	Sequence (5'-3')
SSIIIa-A	C2074	Wildtype	TTAGTGTCCCTGAGCAAAAGC
		Mutant	TTAGTGTCCCTGAGCAAAAGT
		Common	ACGGCCGAAAGATCTTGACTAT
SSIIIa-B	C0905	Wildtype	GAGTGCTTGCCATTCTGGATTG
		Mutant	GAGTGCTTGCCATTCTGGATTA
		Common	GGAGTAGCTTATTTGCTGCATTAG
SSIIIa-D	C0291	Wildtype	TTGTTCTTCAACCCTGACAAGTCG
		Mutant	TTGTTCTTCAACCCTGACAAGTCA
		Common	CAGGTGTAAGTTTGAATCCTGAGC

Table S2. *Number of mutant and sibling control plants selected from segregating F_2 populations.*

Genotype	Plants selected
Sibling control	8
ssIIIa-A	10
ssIIIa-B	13
ssIIIa-D	15
ssIIIa-AB	10
ssIIIa-AD	5
ssIIIa-BD	10
ssIIIa-ABD	11

Table S3. *Genome-specific sequencing primers used to confirm presence or absence of each mutant ssIIIa allele. Line is the mutant number in the TILLING population (e.g., C2074 = Cadenza 2074).*

**Gene**	**Line**	**Amplicon size (bp)**	**Primer**	**Sequence (5'-3')**	**Tm (°C)**
SSIIIa-1A	C2074	204	Forward	ACCAAGACAAAACCAATCAATCA	60
			Reverse	ACGGCCGAAAGATCTTGACTAT	61
SSIIIa-1B	C0905	857	Forward	GGAGTAGCTTATTTGCTGCATTA	58
			Reverse	ATCGTTCACAAGGGATGATGG	62
SSIIIa-1D	C0291	730	Forward	TGGCTGATCAAGACGCAG	60
			Reverse	AATTTTACATGAAGCACATCCTC	57

Table S4. *Mean and standard deviation (in parentheses) of all outcomes across genotypes.*

	Sibling Control	A	B	D	AB	AD	BD	ABD
Resistant Starch (g/100g flour)	2.86 (1.15)	2.40 (0.72)	2.59 (0.89)	2.59 (1.28)	3.76 (0.50)	3.75 (0.50)	3.98 (1.65)	6.01 (0.61)
Resistant Starch (g/100g starch)	0.69 (0.11)	0.72 (0.16)	0.80 (0.13)	0.47 (0.09)	0.87 (0.20)	0.78 (0.27)	0.69 (0.06)	1.70 (0.59)
Amylose (%) (using iodine binding)	26 (3.2)	26.7 (1.8)	27.3 (1.3)	25.5 (2.8)	28.9 (4.7)	26.4 (3.1)	26.3 (2.4)	35.1 (0.56)
Starch (%)	68.1 (5.6)	65.8 (4.7)	64.4 (2.5)	64.4 (2.1)	63.7 (2.5)	61.4 (3)	59 (2.9)	56.1 (2.7)
Starch per grain (mg)	28.7 (1.1)	27 (3.2)	27.9 (3)	27.2 (2.6)	26.6 (2.4)	26.9 (3.9)	23.3 (2.3)	20.3 (2.2)
Grain area (mm^2)	17.6 (0.57)	17.4 (1)	18.5 (1.1)	17.3 (1.1)	18 (0.81)	18.3 (0.88)	17.2 (1.1)	16.7 (0.52)
Grain width (mm)	3.38 (0.087)	3.35 (0.077)	3.46 (0.078)	3.35 (0.12)	3.38 (0.074)	3.43 (0.12)	3.27 (0.14)	3.2 (0.1)
Thousand grain weight (g)	42.3 (2.8)	41.3 (3)	43.5 (3.4)	40.8 (4.2)	41.8 (2)	43.7 (4.5)	39.4 (3.5)	37.7 (3)
Grain length (mm)	6.52 (0.14)	6.48 (0.29)	6.63 (0.3)	6.44 (0.23)	6.56 (0.17)	6.62 (0.16)	6.51 (0.29)	6.48 (0.14)
Proportion of volume as B-type granules	0.367 (0.035)	0.362 (0.025)	0.363 (0.0096)	0.368 (0.048)	0.395 (0.048)	0.364 (0.04)	0.372 (0.035)	0.393 (0.032)
Mean diameter of B-type granules (µm)	5.11 (0.14)	5.11 (0.16)	5.15 (0.19)	5.22 (0.22)	5.22 (0.14)	5.06 (0.32)	4.92 (0.18)	4.97 (0.39)
Mean diameter of A-type granules (µm)	16.7 (0.28)	16.9 (0.66)	17.1 (0.29)	17.4 (0.78)	16.7 (0.58)	16.5 (0.87)	15.6 (0.71)	13.9 (0.75)
Protein (%)	9.0 (0.5)	9.4 (0.9)	8.0 (1.6)	8.1 (2.1)	8.6 (0.5)	9.2 (2.0)	8.9 (1.7)	10.3 (2.1)
Total AXOS	32.7 (3.6)	32.8 (2.4)	34.1 (2.3)	34.0 (1.9)	35.3 (2.7)	35.1 (2.9)	38.2 (3.2)	44.1 (1.9)
Substituted AXOS	11.6 (0.4)	11.7 (0.3)	12.0 (0.9)	12.9 (0.4)	13.2 (1.3)	14.0 (1.1)	14.3 (0.7)	16.7 (0.8)
Unsubstituted AXOS	21.0 (3.3)	21.0 (2.6)	22.1 (1.7)	21.1 (1.7)	22.1 (1.5)	21.2 (2.3)	24.0 (2.6)	27.4 (1.6)
Substituted: unsubstituted AXOS	0.56 (0.09)	0.57 (0.08)	0.54 (0.04)	0.61 (0.05)	0.60 (0.04)	0.67 (0.08)	0.60 (0.05)	0.61 (0.04)
MLG (G3 + G4)	9.1 (1.2)	9.3 (1.1)	9.7 (0.6)	10.8 (0.4)	10.6 (0.6)	10.0 (1.4)	10.6 (0.6)	12.7 (1.5)
G3:G4	2.52 (0.07)	2.53 (0.12)	2.54 (0.08)	2.59 (0.08)	2.63 (0.09)	2.51 (0.09)	2.49 (0.14)	2.50 (0.08)
Height of amylose peak / height of amylopectin peak	0.35 (0.02)	0.35 (0.04)	0.38 (0.04)	0.39 (0.06)	0.49 (0.03)	0.44 (0.03)	0.49 (0.08)	0.63 (0.12)
% Amylose content (using HPLC-SEC)	25.5 (1.7)	25.3 (1.4)	27.0 (2.4)	27.5 (3.0)	29.2 (1.2)	28.9 (1.0)	29.9 (3.0)	33.8 (4.0)
% Long-chain amylopectin as a proportion of all amylopectin	35.5 (2.3)	35.9 (0.7)	37.4 (0.9)	37.0 (1.3)	30.4 (1.3)	29.9 (0.7)	29.6 (0.9)	28.3 (2.1)
Glucose (mg/grain) 25 DAA	0.13 (0.02)	-	-	-	-	-	-	0.18 (0.06)
Fructose (mg/grain) 25 DAA	0.47 (0.23)	-	-	-	-	-	-	0.58 (0.18)
Sucrose (mg/grain) 25 DAA	1.00 (0.06)	-	-	-	-	-	-	3.04 (0.41)
Glucose (mg/grain) Mature	0.07 (0.02)	-	-	-	-	-	-	0.09 (0.01)
Fructose (mg/grain) Mature	0.10 (0.03)	-	-	-	-	-	-	0.18 (0.04)
Sucrose (mg/grain) Mature	0.57 (0.06)	-	-	-	-	-	-	1.09 (0.06)
Enthalpy (J/g)	7.64 (0.51)	-	-	-	-	-	-	7.78 (0.51)
Onset temp (°C)	43.4 (0.86)	-	-	-	-	-	-	42.8 (0.82)
Peak temp (°C)	58.4 (0.76)	-	-	-	-	-	-	61.8 (0.95)
Conclusion temp (°C)	70.5 (1.2)	-	-	-	-	-	-	75.4 (1.9)
Germination Index	0.737 (0.035)	-	-	-	-	-	-	0.693 (0.021)
Hardness	59.6 (9.7)	-	-	-	-	-	-	79 (4.2)

Table S5. *Sample sizes (biological replicates) measured for each outcome.*

	Sibling Control	A	B	D	AB	AD	BD	ABD
Resistant Starch (g/100g flour)	5	5	5	5	5	5	5	5
Resistant Starch (g/100g starch)	5	5	5	5	5	5	5	5
Amylose (%)	5	5	5	5	5	5	5	5
Starch (%)	5	5	5	5	5	5	5	5
Starch per grain (mg)	5	5	5	5	5	5	5	5
Grain area (mm^2)	8	10	13	15	10	5	10	11
Grain width (mm)	8	10	13	15	10	5	10	11
Thousand grain weight (g)	8	10	13	15	10	5	10	11
Grain length (mm)	8	10	13	15	10	5	10	11
Proportion of volume as B-type granules	5	5	4	5	5	5	5	5
Mean diameter of B-type granules (µm)	5	5	4	5	5	5	5	5
Mean diameter of A-type granules (µm)	5	5	4	5	5	5	5	5
Protein (%)	8	10	13	15	10	5	10	11
Total AXOS	5	5	5	5	5	5	5	5
Substituted AXOS	5	5	5	5	5	5	5	5
Unsubstituted AXOS	5	5	5	5	5	5	5	5
MLG (G3 + G4)	5	5	5	5	5	5	5	5
Ratio of SEC peaks	5	5	5	5	5	5	5	4
Ratio of SEC areas 1	5	5	5	5	5	5	5	4
Ratio of SEC areas 2	5	5	5	5	5	5	5	4
Germination Index	5	5	5	5	5	5	5	5
Glucose, Fructose and Sucrose (mg/grain) 25 DAA	3	-	-	-	-	-	-	3
Glucose, Fructose and Sucrose (mg/grain) Mature	3	-	-	-	-	-	-	4
Enthalpy (J/g)	5	-	-	-	-	-	-	5
Onset temp (°C)	5	-	-	-	-	-	-	5
Peak temp (°C)	5	-	-	-	-	-	-	5
Conclusion temp (°C)	5	-	-	-	-	-	-	5
Hardness	5	-	-	-	-	-	-	5

Table S6. *Sugar contents (mg/grain) of triple ssIIIa-ABD mutants and sibling controls at 25 days after anthesis (DAA) and maturity. Values represent the mean and (standard deviation). n = number of biological replicates from independent plants. 'Total' represents the sum of sucrose, glucose and fructose values.*

**25 DAA**	***n***	**Sucrose**	**Glucose**	**Fructose**	**Total**
Sibling control	3	1.00 (0.06)	0.13 (0.02)	0.47 (0.23)	1.60
ssIIIa-ABD	3	3.04 (0.41)	0.18 (0.06)	0.58 (0.18)	3.80
**Mature grains**					
Sibling control	3	0.57 (0.06)	0.07 (0.02)	0.10 (0.03)	0.74
ssIIIa-ABD	4	1.09 (0.06)	0.09 (0.01)	0.18 (0.04)	1.36

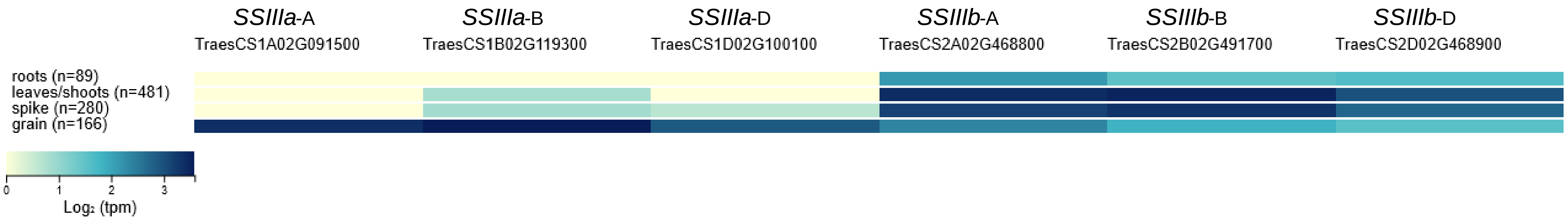

Figure S1. *Expression heatmap of SSIIIa and SSIIIb homoeologues in high level plant tissues. The heatmap was generated by the Wheat Expression browser using the RefSeq1.1 Gene set (http://www.wheat-expression.com/) and data from all studies available in the browser database. Log*$_2$*(tpm) is used as the expression unit; tpm values below 1 were forced to have a log*$_2$ *value of zero since tpm values below 2 are considered as very low expressed genes and log*$_2$ *values of tpm < 1 result in negative expression values.*

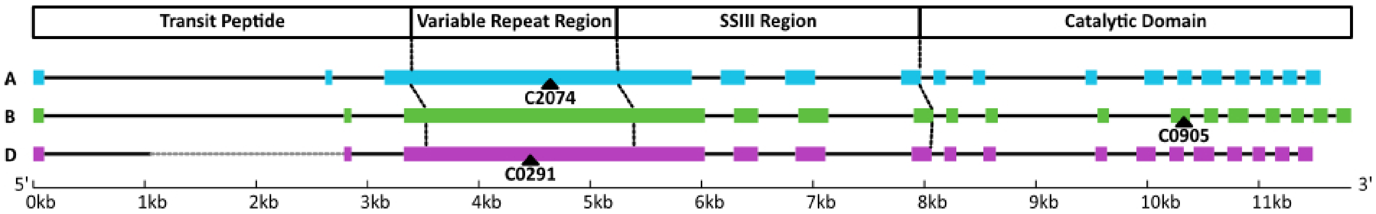

Figure S2. *Gene models for SSIIIa homoeologues and approximate positions of selected mutations. Solid black lines represent introns and coloured bars represent exons. For the ssIIIa-D homoeologue a grey dashed line was inserted to align the exons of the three homoeologues to show that they all have a similar exon-intron structure. The approximate positions of mutations are indicated with a black triangle and labelled with the mutant number in the TILLING population (e.g., C2074 = Cadenza 2074). The approximate positions of corresponding protein domains are based on the defined proteins domains described in Li et al. (2000).*

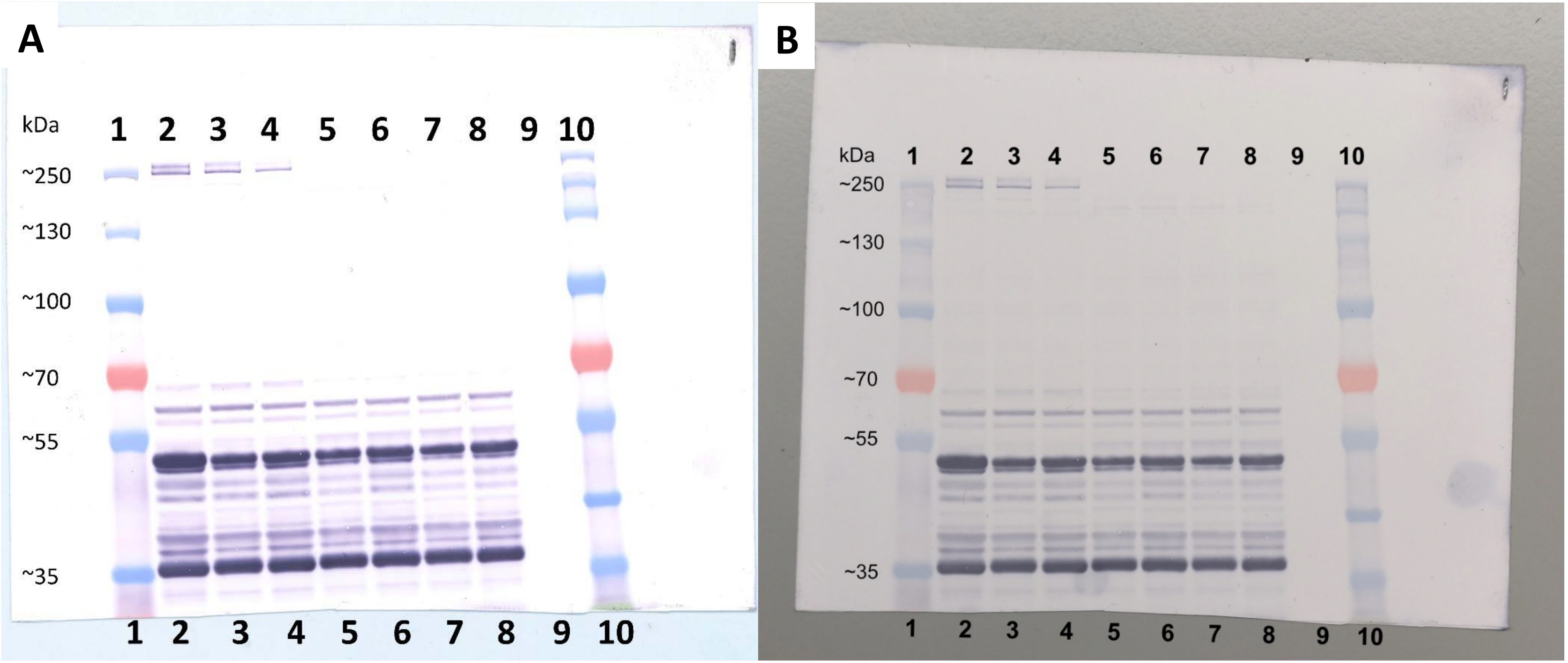

Figure S3. (A) *Original uncropped immunoblot image used to make Figure 1, and* ***(B)*** *image of the same immunoblot with less contrast. Samples loaded in each lane are as follows:*

Lane 1: PageRuler Plus Prestained Protein Ladder (Thermo #26619)

Lanes 2, 3 and 4: sibling control

Lanes 5, 6, 7: ssIIIa-ABD

Lane 8: ssIIIa-ABD (this sample was from a separate experiment)

Lane 9: Blank

Lane 10: Color Prestained Protein Standard (NEB #P7719)

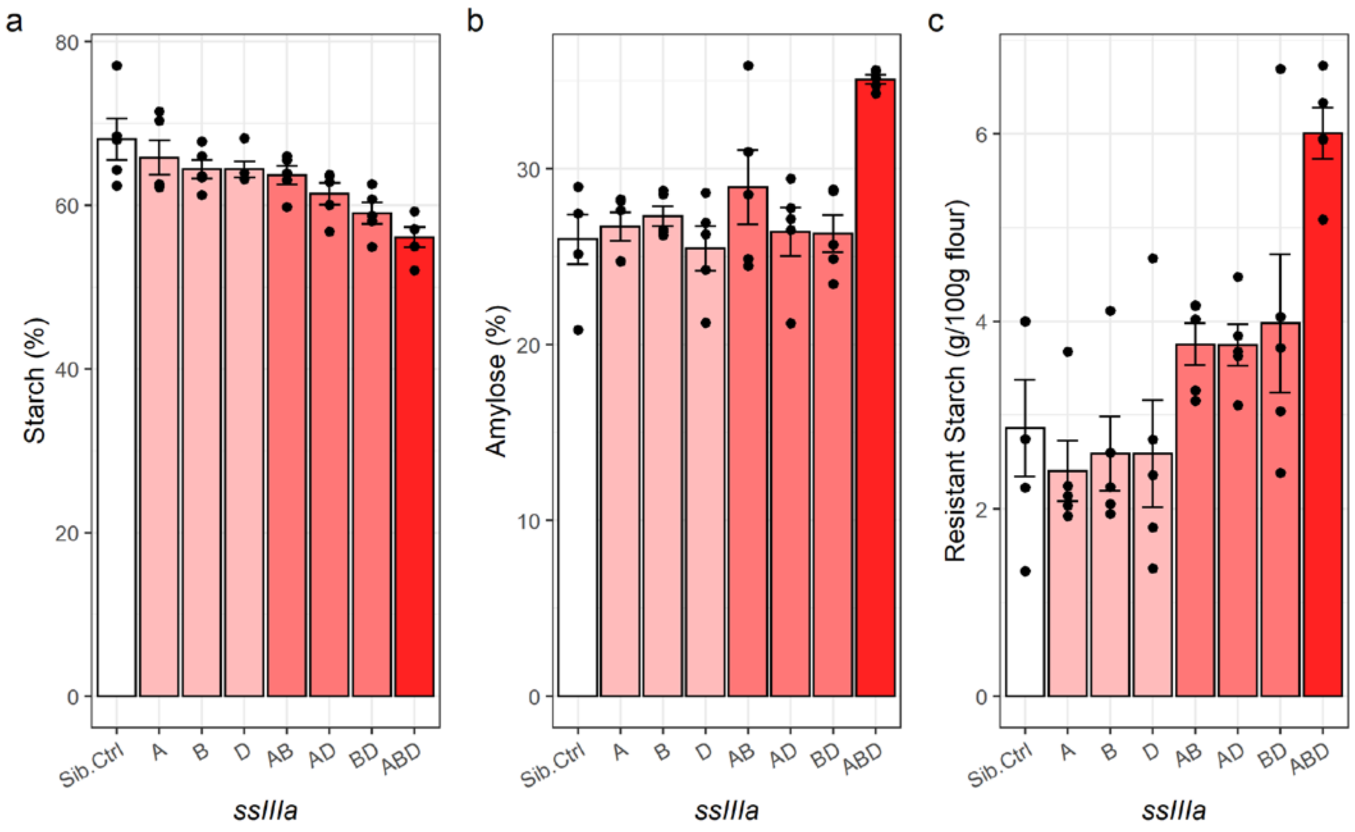

Figure S4. a. *Starch content (%) of wholemeal flour.* ***b.*** *Amylose content (%) of isolated starch (measured by iodine binding method)* ***c.*** *Resistant starch content of wholemeal flour (g/100g of flour). The bars represent the mean ± standard error of each genotype and the dots represent the individual biological replicates (n = 5).*

Regression models

For each set of outcomes, the tables show ‘Comparison of mutants to sibling control’ and for outcomes measured on all genotypes the ‘Comparison of each mutation and interaction effects’.

Two models are estimated for each outcome. The first compares each of seven mutant genotypes to the sibling control. The second simultaneously estimates the average main effect of each mutation, and their synergistic effects measured as interactions.

The three values for each outcome represent (1) regression coefficients (estimates of effects), (2) standard errors of estimates (in parentheses), and (3) *P*-values calculated using models described in the main text. In each case the Sibling control is the reference group.

Grain physical characteristics

		**Grain area (mm^2)**	**Grain width (mm)**	**TGW (g)**	**Grain length (mm)**
Comparison of mutants to sibling	Sibling control	18 (0.33)	3.4 (0.036)	42 (1.2)	6.5 (0.082)
	ssIIIa-A vs control	-0.2 (0.45) p=0.652	-0.028 (0.048) p=0.569	-1 (1.6) p=0.532	-0.033 (0.11) p=0.763
	ssIIIa-B vs control	0.84 (0.42) p=0.051	0.08 (0.046) p=0.086	1.2 (1.5) p=0.44	0.11 (0.1) p=0.283
	ssIIIa-D vs control	-0.3 (0.41) p=0.463	-0.022 (0.045) p=0.622	-1.5 (1.5) p=0.313	-0.081 (0.1) p=0.427
	ssIIIa-AB vs control	0.34 (0.45) p=0.455	0.007 (0.048) p=0.885	-0.48 (1.6) p=0.767	0.039 (0.11) p=0.722
	ssIIIa-AD vs control	0.65 (0.54) p=0.23	0.046 (0.058) p=0.428	1.4 (1.9) p=0.464	0.11 (0.13) p=0.427
	ssIIIa-BD vs control	-0.46 (0.45) p=0.308	-0.11 (0.048) p=0.031	-2.9 (1.6) p=0.071	-0.0031 (0.11) p=0.977
	ssIIIa-ABD vs control	-0.92 (0.44) p=0.04	-0.18 (0.047) <0.001	-4.6 (1.6) p=0.004	-0.042 (0.11) p=0.695
Comparison of each mutation and	Sibling control	18 (0.33)	3.4 (0.036)	42 (1.2)	6.5 (0.082)
	A	-0.2 (0.45) p=0.652	-0.028 (0.048) p=0.569	-1 (1.6) p=0.532	-0.033 (0.11) p=0.763
	B	0.84 (0.42) p=0.051	0.08 (0.046) p=0.086	1.2 (1.5) p=0.44	0.11 (0.1) p=0.283
	D	-0.3 (0.41) p=0.463	-0.022 (0.045) p=0.622	-1.5 (1.5) p=0.313	-0.081 (0.1) p=0.427
	AB interaction	-0.3 (0.6) p=0.614	-0.045 (0.064) p=0.485	-0.65 (2.1) p=0.762	-0.04 (0.15) p=0.786
	AD interaction	1.2 (0.66) p=0.084	0.096 (0.072) p=0.184	3.9 (2.4) p=0.102	0.22 (0.16) p=0.18
	BD interaction	-0.99 (0.57) p=0.086	-0.16 (0.062) p=0.01	-2.6 (2) p=0.207	-0.035 (0.14) p=0.807
	ABD interaction	-1.1 (0.87) p=0.208	-0.097 (0.094) p=0.307	-4 (3.1) p=0.208	-0.19 (0.21) p=0.389

Grain protein content

		Protein (%)
Comparison of mutants to sibling control	Sibling control	9.05 (0.70)
	ssIIIa-A vs control	0.39 (0.99) p=0.70
	ssIIIa-B vs control	-1.00 (0.99) p=0.32
	ssIIIa-D vs control	-0.95 (0.99) p=0.35
	ssIIIa-AB vs control	-0.42 (0.99) p=0.68
	ssIIIa-AD vs control	-0.17 (0.99) p=0.87
	ssIIIa-BD vs control	0.11 (0.99) p=0.91
	ssIIIa-ABD vs control	1.23 (0.99) p=0.22
Comparison of each mutation and synergistic effects	*Sibling control*	9.05 (0.70)
	A	0.39 (0.99) p=0.70
	B	-1.00 (0.99) p=0.32
	D	-0.95 (0.99) p=0.35
	AB interaction	0.20 (1.40) p=0.89
	AD interaction	0.67 (1.40) p=0.64
	BD interaction	1.78 (1.40) p=0.21
	ABD interaction	0.14 (1.98) p=0.95

Non-starch polysaccharides

		Relative peak values			
		**Total AXOS**	**Substituted AXOS**	**Unsubstituted AXOS**	**MLG (G3 + G4)**
Comparison of mutants to sibling control	Sibling control	32.67 (1.19)	11.63 (0.38)	21.04 (1.01)	9.06 (0.45)
	ssIIIa-A vs control	0.10 (1.69) p=0.95	0.11 (0.53) p=0.83	-0.01 (1.43) p=1.00	0.25 (0.64) p=0.70
	ssIIIa-B vs control	1.45 (1.69) p=0.40	0.35 (0.53) p=0.52	1.10 (1.43) p=0.45	0.65 (0.64) p=0.32
	ssIIIa-D vs control	1.30 (1.69) p=0.45	1.26 (0.53) p=0.02	0.04 (1.43) p=0.98	1.69 (0.64) p=0.01
	ssIIIa-AB vs control	2.63 (1.69) p=0.13	1.58 (0.53) p=0.01	1.05 (1.43) p=0.47	1.56 (0.64) p=0.02
	ssIIIa-AD vs control	2.45 (1.69) p=0.16	2.34 (0.53) p<0.001	0.10 (1.43) p=0.94	0.95 (0.64) p=0.15
	ssIIIa-BD vs control	5.56 (1.69) p=0.00	2.62 (0.53) p<0.001	2.94 (1.43) p=0.05	1.54 (0.64) p=0.02
	ssIIIa-ABD vs control	11.46 (1.69) p<0.001	5.09 (0.53) p<0.001	6.37 (1.43) p<0.001	3.61 (0.64) p<0.001
Comparison of each mutation and interaction effects	*Sibling control*	32.67 (1.19)	11.63 (0.38)	21.04 (1.01)	9.06 (0.45)
	A	0.10 (1.69) p=0.95	0.11 (0.53) p=0.83	-0.01 (1.43) p=1.00	0.25 (0.64) p=0.70
	B	1.45 (1.69) p=0.40	0.35 (0.53) p=0.52	1.10 (1.43) p=0.45	0.65 (0.64) p=0.32
	D	1.30 (1.69) p=0.45	1.26 (0.53) p=0.02	0.04 (1.43) p=0.98	1.69 (0.64) p=0.01
	AB interaction	1.08 (2.39) p=0.65	1.13 (0.75) p=0.14	-0.04 (2.02) p=0.98	0.66 (0.91) p=0.48
	AD interaction	1.04 (2.39) p=0.67	0.97 (0.75) p=0.21	0.07 (2.02) p=0.97	-0.99 (0.91) p=0.28
	BD interaction	2.81 (2.39) p=0.25	1.02 (0.75) p=0.19	1.79 (2.02) p=0.38	-0.80 (0.91) p=0.38
	ABD interaction	3.67 (3.38) p=0.29	0.26 (1.06) p=0.81	3.41 (2.86) p=0.24	2.15 (1.29) p=0.10

Starch properties

		Resistant Starch (g/100g flour)	Resistant Starch (g/100g starch)	Amylose (%) (using iodine binding)	Starch (%)	Starch per grain (mg)
Comparison of mutants to sibling control	Sibling control	2.86 (0.45)	0.69 (0.11)	26 (1.2)	68 (1.5)	29 (1.2)
	ssIIIa-A vs control	-0.46 (0.64) p=0.48	0.03 (0.16) p=0.84	0.74 (1.7) p=0.674	-2.2 (2.2) p=0.316	-1.7 (1.7) p=0.312
	ssIIIa-B vs control	-0.27 (0.64) p=0.67	0.11 (0.16) p=0.51	1.3 (1.7) p=0.45	-3.6 (2.2) p=0.104	-0.78 (1.7) p=0.649
	ssIIIa-D vs control	-0.27 (0.64) p=0.67	-0.22 (0.16) p=0.19	-0.48 (1.7) p=0.785	-3.7 (2.2) p=0.101	-1.6 (1.7) p=0.361
	ssIIIa-AB vs control	0.89 (0.64) p=0.16	0.18 (0.16) p=0.26	3 (1.7) p=0.094	-4.4 (2.2) p=0.052	-2.1 (1.7) p=0.221
	ssIIIa-AD vs control	0.89 (0.64) p=0.17	0.10 (0.16) p=0.56	0.47 (1.7) p=0.788	-6.7 (2.2) p=0.005	-1.8 (1.7) p=0.292
	ssIIIa-BD vs control	1.12 (0.64) p=0.08	0.00 (0.16) p=1.00	0.33 (1.7) p=0.848	-9 (2.2) <0.001	-5.4 (1.7) p=0.003
	ssIIIa-ABD vs control	3.15 (0.64) p<0.001	1.01 (0.16) p<0.001	9.1 (1.7) <0.001	-12 (2.2) <0.001	-8.4 (1.7) <0.001
Comparison of each mutation and interaction effects	*Sibling control*	2.86 (0.45)	0.69 (0.11)	26 (1.2)	68 (1.5)	29 (1.2)
	A	-0.46 (0.64) p=0.48	0.03 (0.16) p=0.84	0.74 (1.7) p=0.674	-2.2 (2.2) p=0.316	-1.7 (1.7) p=0.312
	B	-0.27 (0.64) p=0.67	0.11 (0.16) p=0.51	1.3 (1.7) p=0.45	-3.6 (2.2) p=0.104	-0.78 (1.7) p=0.649
	D	-0.27 (0.64) p=0.67	-0.22 (0.16) p=0.19	-0.48 (1.7) p=0.785	-3.7 (2.2) p=0.101	-1.6 (1.7) p=0.361
	AB interaction	1.62 (0.90) p=0.08	0.04 (0.23) p=0.85	0.93 (2.5) p=0.706	1.5 (3.1) p=0.639	0.41 (2.4) p=0.867
	AD interaction	1.62 (0.90) p=0.08	0.28 (0.23) p=0.23	0.21 (2.5) p=0.932	-0.76 (3.1) p=0.806	1.5 (2.4) p=0.537
	BD interaction	1.66 (0.90) p=0.07	0.11 (0.23) p=0.64	-0.52 (2.5) p=0.836	-1.7 (3.1) p=0.588	-3 (2.4) p=0.214
	ABD interaction	-0.75 (1.27) p=0.56	0.66 (0.32) p=0.05	6.9 (3.5) p=0.056	-1.4 (4.4) p=0.75	-3.2 (3.4) p=0.353

Starch granule size distribution

		Proportion of volume as B-type particles	Mean diameter of B-type granules (µm)	Mean diameter of A-type granules (µm)
Comparison of mutants to sibling	Sibling control	0.37 (0.016)	5.1 (0.1)	17 (0.29)
	ssIIIa-A vs control	-0.0042 (0.022) p=0.851	0.0025 (0.15) p=0.986	0.28 (0.42) p=0.509
	ssIIIa-B vs control	-0.0012 (0.023) p=0.959	0.041 (0.16) p=0.797	0.46 (0.44) p=0.309
	ssIIIa-D vs control	0.0051 (0.022) p=0.82	0.11 (0.15) p=0.463	0.71 (0.42) p=0.1
	ssIIIa-AB vs control	0.027 (0.022) p=0.235	0.11 (0.15) p=0.461	-0.013 (0.42) p=0.975
	ssIIIa-AD vs control	-0.01 (0.021) p=0.635	-0.055 (0.15) p=0.711	-0.16 (0.42) p=0.71
	ssIIIa-BD vs control	0.0029 (0.021) p=0.892	-0.19 (0.15) p=0.215	-1 (0.42) p=0.019
	ssIIIa-ABD vs control	0.023 (0.022) p=0.297	-0.14 (0.15) p=0.355	-2.7 (0.42) <0.001
Comparison of each mutation and	*Sibling control*	0.37 (0.016)	5.1 (0.1)	17 (0.29)
	A	-0.0042 (0.022) p=0.851	0.0025 (0.15) p=0.986	0.28 (0.42) p=0.509
	B	-0.0012 (0.023) p=0.959	0.041 (0.16) p=0.797	0.46 (0.44) p=0.309
	D	0.0051 (0.022) p=0.82	0.11 (0.15) p=0.463	0.71 (0.42) p=0.1
	AB interaction	0.032 (0.032) p=0.322	0.067 (0.22) p=0.757	-0.75 (0.61) p=0.227
	AD interaction	-0.011 (0.032) p=0.725	-0.17 (0.21) p=0.429	-1.1 (0.59) p=0.062
	BD interaction	-0.00099 (0.033) p=0.976	-0.34 (0.22) p=0.127	-2.2 (0.61) p=0.001
	ABD interaction	0.0033 (0.046) p=0.942	0.15 (0.3) p=0.63	-0.092 (0.85) p=0.914

Starch chain length distribution

		Height of amylose peak / height of amylopectin peak	% Amylose content (using HPLC-SEC)	% Long-chain amylopectin as a proportion of all amylopectin
Comparison of mutants to sibling control	Sibling control	0.35 (0.03)	25.54 (1.06)	35.51 (0.61)
	ssIIIa-A vs control	0.00 (0.04) p=0.99	-0.19 (1.50) p=0.90	0.39 (0.86) p=0.66
	ssIIIa-B vs control	0.03 (0.04) p=0.44	1.42 (1.50) p=0.35	1.86 (0.86) p=0.04
	ssIIIa-D vs control	0.05 (0.04) p=0.22	1.92 (1.50) p=0.21	1.52 (0.86) p=0.09 +
	ssIIIa-AB vs control	0.14 (0.04) p<0.001	3.71 (1.50) p=0.02	-5.12 (0.86) p<0.001
	ssIIIa-AD vs control	0.09 (0.04) p=0.02	3.38 (1.50) p=0.03	-5.58 (0.86) p<0.001
	ssIIIa-BD vs control	0.14 (0.04) p<0.001	4.36 (1.50) p=0.01	-5.96 (0.86) p<0.001
	ssIIIa-ABD vs control	0.28 (0.04) p<0.001	8.25 (1.59) p<0.001	-7.22 (0.92 p<0.001
Comparison of each mutation and interaction effects	Sibling control	0.35 (0.03)	25.54 (1.06)	35.51 (0.61)
	A	0.00 (0.04) p=0.99	-0.19 (1.50) p=0.90	0.39 (0.86) p=0.66
	B	0.03 (0.04) p=0.44	1.42 (1.50) p=0.35	1.86 (0.86) p=0.04
	D	0.05 (0.04) p=0.22	1.92 (1.50) p=0.21	1.52 (0.86) p=0.09
	AB interaction	0.11 (0.05) p=0.04 *	2.48 (2.12) p=0.25	-7.37 (1.22) p<0.001
	AD interaction	0.05 (0.05) p=0.38	1.65 (2.12) p=0.44	-7.48 (1.22) p<0.001
	BD interaction	0.06 (0.05) p=0.22	1.02 (2.12) p=0.63	-9.34 (1.22) p<0.001
	ABD interaction	-0.02 (0.07) p=0.80	-0.05 (3.04) p=0.99	13.21 (1.75) p<0.001

Differential scanning calorimetry parameters

		Enthalpy (J/g)	Onset temp (°C)	Peak temp (°C)	Conclusion temp (°C)
Comparison of mutants to sibling control	Sibling control	7.6 (0.23)	43 (0.38)	58 (0.39)	70 (0.71)
	ssIIIa-ABD vs control	0.14 (0.32) p=0.678	-0.68 (0.53) p=0.239	3.4 (0.55) p<0.001	4.9 (1) p=0.001

Soluble sugars

Estimated ratio of sugars (mg/grain) between triple mutant and sibling control (ratio, 95% confidence intervals, p-value)

	Fructose	Sucrose	Glucose	Total sugars
**25DAA**	1.31 (0.69-2.49) p=0.369	2.99 (2.19-4.08) p<0.001	1.36 (0.84-2.22) p=0.181	2.39 (1.81-3.16) p<0.001
**Mature**	1.88 (1.03-3.42) p=0.042	1.93 (1.44-2.58) p=0.001	1.25 (0.79-1.97) p=0.296	1.85 (1.42-2.39) p<0.001

Germination index and hardness

		Hardness
Comparison of mutants to sibling control	Sibling control	60 (3.3) <0.001
	ssIIIa-ABD vs control	19 (4.7) p=0.003

Method for HPLC-SEC

Equipped with refractive index detector (RI), autosampler (40 °C), and column heater (90°C), a Waters Alliance e2695 HPLC (Milford, US) was used for peak resolution by size exclusion. An isocratic mobile phase of DMSO with 0.5% LiBr (w/w) at 0.500 mL/min flow rate and a stationary phase was in series beginning with a guard column (8x50mm, GRAM; Polymer Standard Service, Mainz, DE) and two analytical columns in series (10um; 300 Å followed by 30 Å; 8x300mm, GRAM; Polymer Standard Service, Mainz, DE). Total run time was 65 minutes and injection volume for each sample was 50 µL. Calibration curves were generated using pullulan standards (PSS-pulkit, Polymer Standard Service, Mainz, DE) with peak molecular weights ranging from 342 to 708,000 Da and correlation coefficients of R2 = 0.9993 ± 0.0005. Purified starch was solubilized and debranched enzymatically using methods adapted from Perez-Moral, et al. [1]. Debranched starch samples were solubilized at a concentration of 8mg/mL and for standards at 2mg/mL with DMSO containing 0.5% LiBr (w/w). All samples and standards were vortexed and stored at 80°C overnight prior to analysis. The relationship between elution volume and hydrodynamic radius (Vh) for the linear glucans was determined using calibration curves described similarly by Cave, et al. [2]. The debranched starch samples RI elution profiles were converted to SEC weight distributions as described in detail by Perez-Moral, et al. [1].

1 Perez-Moral, N., Plankeele, J.-M., Domoney, C. & Warren, F. J. Ultra-high performance liquid chromatography-size exclusion chromatography (UPLC-SEC) as an efficient tool for the rapid and highly informative characterisation of biopolymers. *Carbohydrate polymers* **196**, 422-426, doi:10.1016/j.carbpol.2018.05.049 (2018).

2 Cave, R. A., Seabrook, S. A., Gidley, M. J. & Gilbert, R. G. Characterization of Starch by Size-Exclusion Chromatography: The Limitations Imposed by Shear Scission. *Biomacromolecules* **10**, 2245-2253, doi:10.1021/bm900426n (2009).
